# Unveiling Phytoplankton Diversity: Taxonomy, Functional Groups, and Environmental Drivers in North China Lakes

**DOI:** 10.1002/ece3.70656

**Published:** 2024-12-03

**Authors:** Wei Wang, Hanjie Huang, Zhongshi He, Guotao Zhang, Junping Lv, Qi Liu, Fangru Nan, Xudong Liu, Shulian Xie, Jia Feng

**Affiliations:** ^1^ School of Life Science Shanxi University Taiyuan China; ^2^ Institute of Marine and Environmental Technology University of Maryland Center for Environmental Science Baltimore Maryland USA

**Keywords:** driving factors, lake or reservoir water bodies, North China, phytoplankton functional groups, phytoplankton taxonomy composition

## Abstract

Functional groups (FGs) represent a classification scheme designed to study the ecological adaptations of phytoplankton. However, FG dynamics studies in phytoplankton are often conducted independent of taxonomic studies, so the factors influencing community dynamics have not been sufficiently investigated or compared between the two classification systems. In this study, we compared the intricate relationship between taxonomic and FG compositions in North China lakes and delve into the key environmental drivers shaping phytoplankton community dynamics. This investigation revealed that taxonomic and FG classifications exhibit high qualitative and quantitative similarities in the community structure. Environmental drivers had a stronger influence on the FG structure than taxonomic composition, indicating that the FG classification does not result in the loss of ecological information regarding the community structure, even with the reduced number of grouping units. Indeed, it was evident that FGs contained a larger quantity of ecological information. These conclusions were further verified using lakes in eastern China. Additionally, we found that climatic–geographical factors usually exerted indirect influences, by altering water chemistry, while water chemical factors had more direct and stronger influences. The combined effects of both types of environmental factors had a greater impact on the phytoplankton FG structure than on taxonomic composition. In conclusion, we believe that an in‐depth study of FGs will better focus on the ecological characteristics of phytoplankton, while also avoiding the need for extensive species identification.

## Graphical abstract

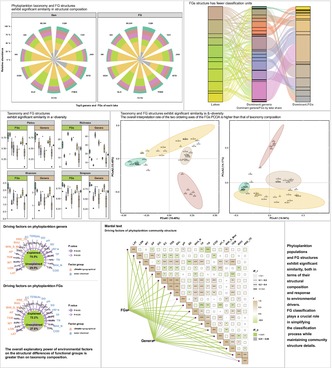

## Abbreviations

**Concepts**
FG: function group

**Sampling Sites**
BYD: Baiyangdian Lake
CQH: Chongqing Lake
DH: Daihai Lake
DLH: Dongli Lake
FHEK: Fenhe Reservoir II
GGH: Guanguang Lake
HH: Haihe River
JHH: Jinhai Lake
WLSH: Wuliangsuhai Lake
YCYH: Yuncheng Salt Lake
YHY: Daming Lake in the Summer Palace

**Environmental Factors**
AP: air pressure
Chl_a: chlorophyll‐a concentration
COD_Mn_
: permanganate index
DO: dissolved oxygen
EC: conductivity
LAT: latitude
LON: longitude
NH_4_^+^‐N: ammonia nitrogen concentration
PRE_7D: precipitation
RHU: relative humidity
SAL: salinity
SD: transparency
TDS: total dissolved solids concentration
TEM: average air temperature
TN: total nitrogen concentration
TP: total phosphorus concentration
WIN_S_Max: maximum wind speed
WT: water temperature.

## 1 Introduction

Phytoplankton, as primary producers in aquatic ecosystems, hold a pivotal position in the food chain, driving energy flow and material cycling (Neri et al. 2023). Their vast diversity and abundance underscore their significance among aquatic primary producers. Due to their tiny size and predominantly unicellular nature, phytoplankton exhibit exquisite sensitivity to environmental variations (Devlin et al. 2019). Extensive research has revealed that alterations in environmental parameters such as water temperature (WT; Mousing, Ellegaard, and Richardson 2014), pH (Dutkiewicz et al. 2015) and nutrient levels (Liu et al. 2007) can profoundly influence phytoplankton community composition. Notably, WT and nutrients have emerged as pivotal determinants of the phytoplankton community structure (Zhang et al. 2018). Consequently, the structure of phytoplankton communities has evolved into a crucial indicator of water quality and eutrophication levels (He et al. 2023).

Traditional classification of the algal community structure relies heavily on morphological characteristics (Fang et al. 2022). While this approach offers precision, manipulating minute individual phytoplankton algal cells poses inherent complexities. Furthermore, the sheer abundance of phytoplankton species translates into substantial workloads, challenging researchers' core competencies (Titilade and Olalekan 2015). Additionally, the susceptibility of phytoplankton community compositions to environmental fluctuations render them nearly impossible to predict across diverse habitats. Consequently, conventional biological methods often fall short in providing an accurate portrayal of aquatic ecosystem health (Reynolds 1980). It is important to note that various environmental factors coexist, and their primary influences can differ from one habitat to another. The interplay between hydrochemical, climatic, hydrodynamic, and food chain factors complicates the precise assessment of the prevailing phytoplankton species within communities. This task is made even more challenging when rigorous preparatory groundwork is not undertaken, and well‐established theoretical models are not available (Yang et al. 2019; Liu et al. 2023).

Given the evolving landscape of scientific research, an increasing number of scholars have recognized this limitation. As a result, some experts have introduced the concept of “phytoplankton functional groups (FGs).” Phytoplankton FGs are classified based on physiological and growth characteristics of these organisms, considering their survival environments and ecological adaptations (Hu Ren, Yuqian, and Boping 2015). Taxa sharing adaptive traits that enable them to thrive or coexist within the same habitat are systematically grouped. Since the initial proposal and utilization of FGs by Reynolds (1980), collaborative efforts have refined and expanded the concept of FG classification. As of September 2023, this classification has evolved from the original 34 species to encompass 39 distinct FGs (Borics et al. 2007). Furthermore, the categorization of “Morpho‐Functional Groups (MFG)” (Salmaso and Padisák 2007) and “Morphology‐Based Functional Groups (MBFG)” (Kruk et al. 2010) categorizations have been meticulously developed. These classifications rely on easy‐to‐understand criteria to classify phytoplankton into several different taxa by means of simple, easily recognizable characteristics (Ma et al. 2022).

The research pertaining to FGs has reached a relatively mature stage of development. A considerable body of research has examined the dynamics of phytoplankton FG community structure and the factors that drive it in a number of lakes (Yang et al. 2014). Some such studies have simultaneously analyzed phytoplankton taxonomic composition (Yan et al. 2023), but the two methodologies remain largely distinct, and no comparative analysis of their results under identical conditions has been conducted (Chen et al. 2019). Concurrently, there is a paucity of research examining the dynamics of phytoplankton FGs on larger scales.

Taking 11 lakes in North China as samples, this research leveraged the concept of phytoplankton FGs to simplify taxonomy while also exploring the intricate interplay among environmental factors affecting algal community structures. We chose to study the FG structure instead of MFG and MBFG because the concept of FGs was the first to be proposed and therefore has undergone the most extensive development and has the best framework. Furthermore, the data from eight lakes in East China were incorporated in the final section, reinforcing the conclusions and broadening the scope of the analysis. We aimed to explore: (1) the similarities and differences in the phytoplankton community structure under different classification principles, particularly regarding taxonomy construction and FGs; and (2) the influence of environmental factors on the phytoplankton community structure.

## 2 Materials and Methods

### 2.1 Sampling Procedure

North China, spanning an area of approximately 870,000 km^2^, encompasses Beijing, Tianjin, Hebei Province, Shanxi Province, and a section of the Inner Mongolia Autonomous Region. The mean annual temperatures range from 8°C to 13°C, while annual precipitation totals range from < 400 to almost 1000 mm. Importantly, North China's water systems are less intricate compared to their southern counterparts (such as East China) (Meng, Zhang, and Shan 2021). Consequently, the lakes and reservoirs here tend to be relatively small, often covering areas of < 100 km^2^. These lakes contribute to regional tourism, agriculture, and fisheries and play pivotal roles in the regional water cycle and climate regulation. Therefore, the safeguard and sustainable harnessing these water bodies are imperative. The comprehensive study, utilization, and conservation of water quality and plankton community structures in these lakes serve as the foundational steps in this ongoing effort.

The total area of East China is 834,300 km^2^, representing 8.7% of the total land area of China. With a favorable climate, abundant produce, and a relatively dense water network, this region has one of the fastest growing economies in China (Li et al. 2019; Zhao, Liu, and Luo 2020). It is also important to note that East China is of a comparable size to North China, despite exhibiting markedly disparate climatic and hydrological characteristics. This makes it particularly well‐suited to comparative studies with North China.

**FIGURE 1:**
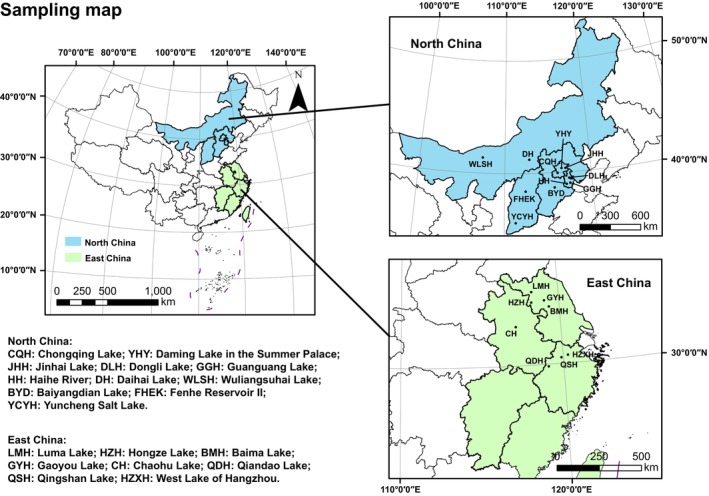
Sampling sites in the current study (2‐column fitting image).

A total of 11 lakes or reservoirs were chosen for sampling from different provinces and cities in the North China region. At least 1–2 lakes or reservoirs were selected from each province and municipality, which could cover most of the North China region (Figure 1). Eight lakes were also selected in East China to supplement the analysis. Detailed information about lakes can be found in Table S1.

### 2.2 Measurement of Physical and Chemical Indicators

WT, dissolved oxygen (DO), conductivity (EC), total dissolved solid concentration (TDS), salinity (SAL), and pH were measured in the field using the YSI ProPlus (YSI Ins, Yellow Springs, OH, USA). Transparency (SD) was measured using a Saybolt disk. Total nitrogen concentration (TN), total phosphorus concentration (TP), ammonia nitrogen concentration (NH_4_^+^_N), permanganate index (COD_Mn_), and chlorophyll‐a concentration (Chl_a) were determined by referring to the national standard method (Yang et al. 2023). Air pressure (AP), maximum wind speed (WIN_S_Max), average air temperature (TEM), relative humidity (RHU), and precipitation (PRE_7D) data were retrieved from the National Meteorological Science Data Center (http://data.cma.cn/).

All environmental factor measurements are detailed in Figure S1, Table S2 (Lake mean), and Table Metadata1 (Samples Metadata). All environmental factor data were categorized into two distinct groups based on indicator type. Group X1 encompasses climatic–geographical factors, including latitude, longitude, WT, TEM, atmospheric pressure, WIN_S_Max, RHU, and PRE_7D. Group X2 encompasses water chemical factors, including EC, total dissolved solids, SAL, DO, pH, transparency, chlorophyll‐a concentration, TP, TN, ammonia nitrogen, and permanganate index.

### 2.3 Collection and Analysis of Biological Samples

Samples were collected from each lake at a minimum of three different distances from the shore: less than 5 m from shore, more than 20 m from shore, and in the center of the lake. We aliquoted and fully mixed five water samples taken from the same offshore distance, but with lateral distances > 10 m, into one water sample. This was done to eliminate sampling errors as much as possible. At each sampling site, we collected phytoplankton samples, including both quantitative and qualitative samples. We completed a phytoplankton sampling record form for each site. Quantitative samples were collected before qualitative samples. A water sampler was used to collect 1 L of water into a sampling bottle at 0.5 m below the water surface, and then Lugol's fixative was added immediately at a fixation ratio of 50:1. During quantitative sample collection, some space was left in the sample bottle between the liquid level and the cap to allow for shaking. In lakes, when phytoplankton such as cyanobacteria float on the water's surface or are distributed in patches or strips, it is important to collect water samples from the densest area of the algae as a peak reference. Qualitative samples were collected by dragging a 25‐gauge plankton net in a “8” pattern at a speed of 20–30 cm/s for 1–3 min from the surface to a depth of 0.5 m. The samples were then collected in sampling bottles and fixed by adding Lugol's fixative in the same proportion (Zhao et al. 2023).

Upon returning to the laboratory, the quantitative samples were thoroughly shaken and poured into the concentrating device. The mixture was then left to stand at room temperature for 24–48 h. The supernatant was extracted using a siphon. The concentration device was rinsed with a small quantity of supernatant solution on three occasions during the process. The precipitate and rinse water were collected together, and the volume was increased to 50 mL.

For qualitative analysis, a micropipette was used to extract approximately 60 μL of the sample from the bottom of the sample bottle for observation under a compound microscope. Each sample was observed three times.

For quantitative analyses, the samples were thoroughly shaken before counting under a microscope. Then, 0.1 mL of the mixed samples was aspirated with a pipette and injected into the phytoplankton counting frame. The frame was covered with a coverslip and left for a few moments to allow bubbles to escape before observing the samples. Finally, five counting cells were randomly selected to count the phytoplankton. Filamentous and spherical‐like groups were estimated by the cell number. The algal cell concentration (cell/L) in the water column of the sample site was calculated based on the cell count. We mainly consulted *the Atlas of Freshwater Microfauna and Benthos* and *the Atlas of Common Aquatic Organisms in Chinese Watersheds* for the taxonomic identification of algae species.

### 2.4 Analysis of Dominant Genera and FGs

Dominant genera were determined using the index of dominance (*Yi*) described by McNaughton (1967) and calculated using the following formula:

(1) $$Y_{i}=\frac{n_{i}}{N}f_{i}$$

where *Y*_*i*_: is the dominance degree of the i‐th phytoplankton species; *n*_*i*_ is the number of individuals of the *i*‐th phytoplankton species; *N* is the total number of individuals of phytoplankton in the sample site; and *f*_*i*_ is the frequency of occurrence of the genus in the sample site.

It is considered to be a dominant species when *Y*_*i*_ ≥ 0.02 and is recognized to be an absolute dominant species when *Y*_*i*_ > 0.1 (Habib, Tippett, and Murphy 1997).

The division of FGs mainly refers to Chen et al. (2019), Xiang et al. (2017), and Zhao et al. (2023). Then, as described by Huang et al. (2015), FGs comprising more than 10% of the total are deemed dominant FGs.

### 2.5 Data Analysis

All analyses were conducted using R4.2.0. Abundance and richness were analyzed, where abundance refers to the number of all phytoplankton individuals in a sample site, and richness stands for the number of phytoplankton species. The Shannon_H index, the Simpson_1‐D index, and the Pielou‐index were calculated (Xu et al. 2023) using the *vegan* package (v2.6‐8, https://CRAN.R‐project.org/package=vegan).

Principal Coordinate Analysis (PCoA) was employed to identify spatial differences in phytoplankton FGs. Redundancy Analysis (RDA) was utilized to analyze the connection between communities and environmental factors and then to determine the relative contributions of environmental factors to changes in the composition of phytoplankton communities and FGs. The Bray–Curtis distances for each sample point group were calculated using the *vegan* package, and subsequently, the *ade4* package (v1.7‐22, https://CRAN.R‐project.org/package=ade4) was used to perform PCoA sorting on the distance matrix, extracting the first two sorting axes for visualization. The standardized community and environmental matrices were subjected to RDA sorting using the “rda” function of the *vegan* package. By setting the parameter “by” can be used anova.cca function to test the significance of the RDA results for global or sorted axes. The test results were significant at *p* < 0.05, and the first two sorting axes were extracted for visualization. Stepwise regression analysis devoid of covariates was performed with the assistance of SPSS 26 to identify crucial environmental factors.

The psych package (2.4.6.26, https://CRAN.R‐project.org/package=psych) was used to calculate correlations and visualize them in PowerPoint. Other results were visualized using the ggplot2 package (3.5.1, https://CRAN.R‐project.org/package=ggplot2).

The aforementioned steps, namely, PCoA, RDA, Envfit test, and correlation analysis, were also employed for the analysis of the sample points in East China. Furthermore, the piecewiseSEM (v.2.3.0.1, https://CRAN.R‐project.org/package=piecewiseSEM) package was employed to make the separate structural equation models of the two regions. A comparison of the structural drivers of phytoplankton communities in the two regions was undertaken. Before applying the equation models, the matrices were first downscaled using PCA analysis. The first principal component, which contained the most information about the matrix, was then used as the eigenvector for the structural equation model.

## 3 Results

### 3.1 Comparison of the Community Structure for Taxonomy and FG Composition

**FIGURE 2:**
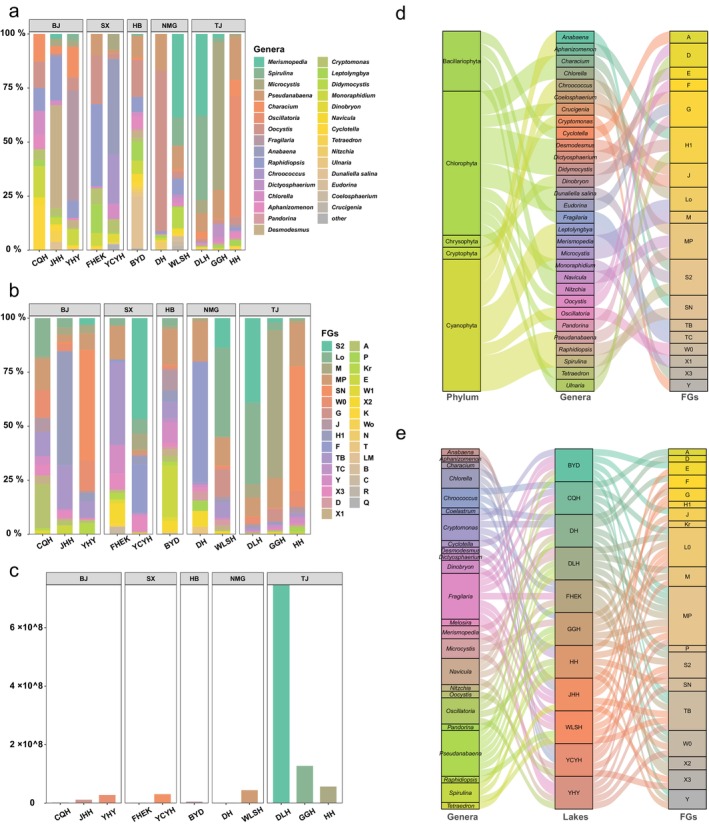
Taxonomy composition of planktonic algae in each lake. (a) The taxonomy composition and relative abundance in each lake. (b) Specific FG structure in each lake. (c) Absolute abundance (cell density) of planktonic algae (unit: cell/L). (d) The classification relationships among the top 30 algae species shared in lakes. (e) The inclusionary relationships between the top 5 genera, FGs, and the lakes from which the samples were obtained. BJ, Beijing City; HB, Hebei Province; NMG, Inner Mongolia Autonomous Region; SX, Shanxi Province; TJ, Tianjin Province.

A combined total of 81 genera of phytoplankton belonging to seven phyla were detected across 11 lakes or reservoirs. Phytoplankton were further classified into 30 FGs. In addition, the taxonomic composition of algae exhibited notable variability among the surveyed lakes (Figure 2a). The top five genera collectively constituted over 60% of the total planktonic algae across all lakes, with this ratio exceeding 80% in the majority of instances (Figure S2, Table S3). Figure 2e provides a comprehensive listing of the 24 genera that appeared in the top 5 in most abundant planktonic algae across in all respective lakes. For more detailed information, see Note S2, and the raw data of the community structure is recorded in Table Metadata2.

**FIGURE 3:**
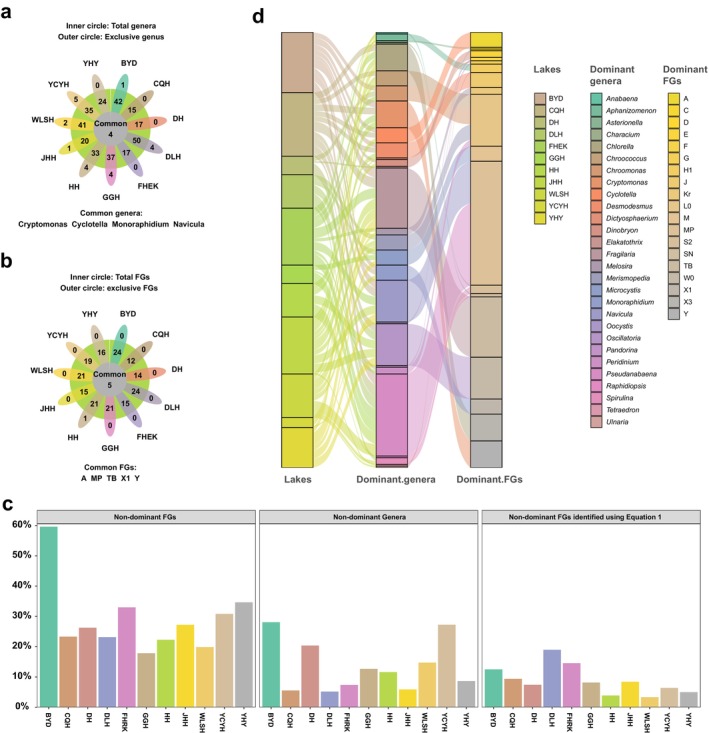
Disparities in phytoplankton genera and FG structure across sampling lakes. (a) Taxonomy composition. (b) FG structure. (c) Relative abundance of non‐dominant genera or FGs in each lake. (d) Dominant genera and FGs in each lake. *Y*_*i*_ FGs: denotes the assessment of dominant functional groups based on dominance (2‐column fitting image).

Interestingly, intriguing parallels emerged in the analyses of FG structures (Figure 2b), where the majority of the top five FGs per lake exhibited higher memberships than the top five genera (Figure S2, Table S4). Nineteen species were consistently presented in the top five FGs across all lakes (Figure 2e). Furthermore, the dominant genera or FGs were generally consistent across freshwater, maintaining robust presence, while non‐dominant genera or FGs typically constituted less than 30% of the total. As depicted in Figure 3, lakes characterized by higher abundances of dominant genera also tended to exhibit greater proportions of dominant FGs, albeit still falling short in the number of dominant genera observed (Figure 3d). Notably, each lake consistently featured at least three dominant genera, while the number of dominant FGs typically remained below three. Dominant FGs in each lake also accounted for a larger proportion than the dominant genus (Figure S3).

**FIGURE 4:**
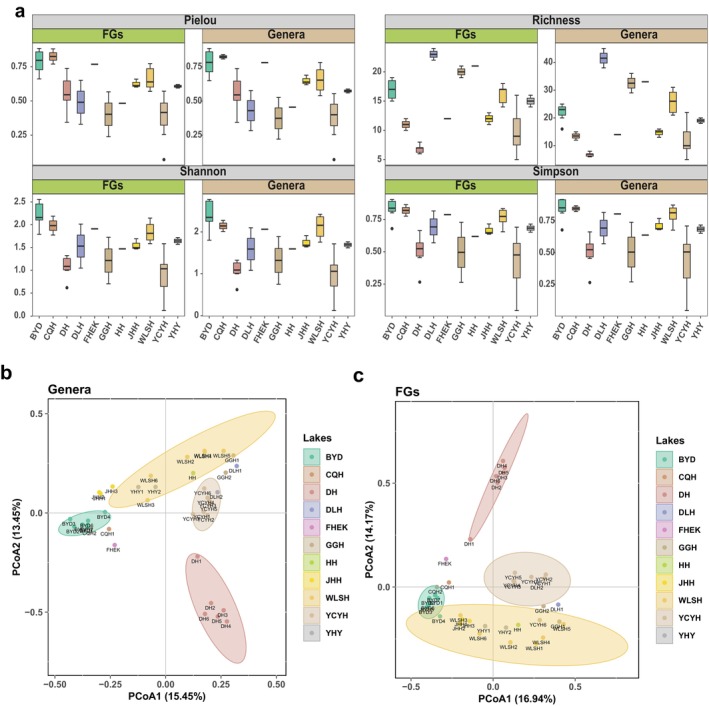
Diversity assessment of lake communities. (a) Alpha diversity. (b) Principal coordinates analysis (PCoA) based on taxonomy composition, and (c) PCoA based on the FG structure (2‐column fitting image).

The α‐diversity of the taxonomic composition, calculated based on species constructions and FGs, also showed high similarity. Daihai Lake had the lowest species diversity, reflected in both low diversity indexes and FGs. Other lakes demonstrated remarkable similarity between the two divisions, in terms of both values and trends (Figure 4a). For both classification methods (index‐FG and index‐G), the four α‐diversity indexes were similar: regardless of whether taxonomic composition or FG structure was considered, DLH Lake exhibited the highest richness, while DH Lake had the lowest. However, the absolute Richness‐FG value in DLH Lake was significantly lower than Richness‐G, whereas in DH Lake, the values were equal (DLH: 23 < 41.5, DH: 6.83 = 6.83). Similar trends were observed for other indices. Importantly, the FGs had greater explanatory power while employing fewer taxonomic units. The PCoA results further corroborated these findings, with the first and second ordering axes of the PCoA for FG analysis explaining 16.94% and 14.17% of the variance, respectively—modestly surpassing the 15.45% and 13.45% explained by the population structure. These outcomes underscored the advantages of employing FGs in classification, providing a more comprehensive and informative overview of population structure.

### 3.2 Driving Factors of Taxonomic Composition and FG Structure

We further conducted an in‐depth exploration of the correlations between taxonomic composition, FG structure, and environmental factors (Figures S4–S6). Chl_a demonstrated significant (*p* < 0.05) positive correlations with the majority of dominant genera and FGs. Furthermore, a notable trend emerged, revealing positive correlations between nutrient salt concentration and planktonic algal density, while precipitation, EC, and SAL consistently demonstrated negative correlations with planktonic algal density. Importantly, there was a prevalent positive and, in some cases, significant (*p* < 0.05) correlation between planktonic algal density and both measured WT at the time of sampling and the average temperature over 7 days. However, instances of negative correlations, and even significant (*p* < 0.05) negative correlations, were observed, resembling patterns seen with latitude and longitude. See Note S3 for a more detailed description. This suggested that there are temporal and spatial influences on the taxonomy and FG communities of phytoplankton. These spatio‐temporal dynamics of the community structure were reflected in both taxonomy and FG structure, with stronger correlations between FGs and environmental factors (Figure S4).

**FIGURE 5:**
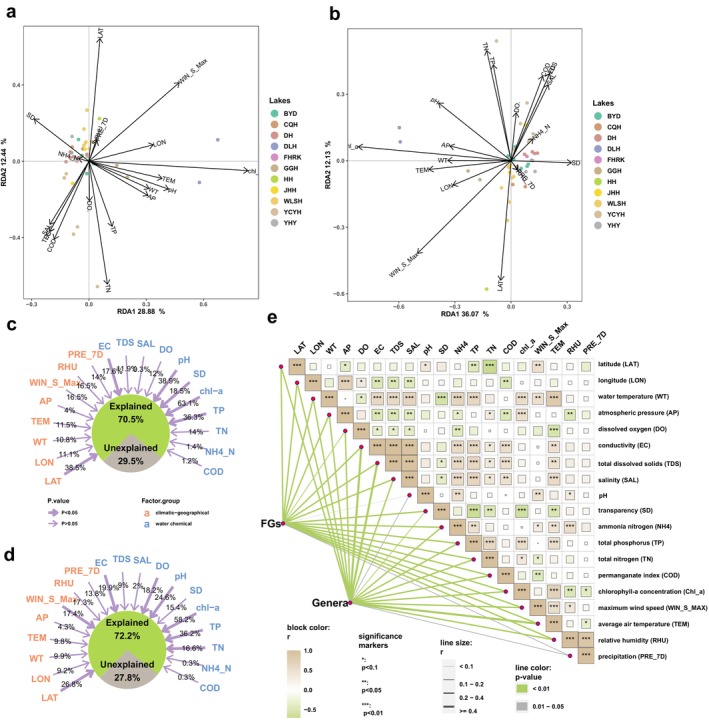
Analysis of the driving factors of phytoplankton genera and FG structure. (a) RDA of taxonomy composition, (b) RDA of FGs, (c, d) individual explanatory power of environmental factors on the structure of phytoplankton communities, and (e) Mantel test (2‐column fitting image).

RDA was employed to investigate the influence of environmental factors on taxonomy and FG structure (Figure 5). The findings revealed discernible impacts of environmental factors on taxonomic composition, where the first and second ordination axes account for 28.88% and 12.44% of the variability, respectively (Figure 5a). Likewise, the impacts of environmental factors on the FG structure were evident, as the first and second ordination axes explained 36.07% and 12.13% of the effects, respectively (Figure 5b). The RDA model for FG composition had a higher rate of explanation. Furthermore, the first ordination axis highlighted the prominence of climatic–geographic factors, with TEM, longitude, WT, and atmospheric pressure assuming more advanced positions, while the second axis emphasized the significance of water chemistry factors, where TP, TN, permanganate index, and pH occupied top positions in both RDA analyses (Table S5).

Figure 5c,d shows detailed analyses of the total explanatory power of environmental factors for the variations in FGs and taxonomic structures, revealing that environmental factors played larger roles in explaining differences in FG differences (72.2%) than taxonomic structure (70.5%). This observation reinforced our assertion that FG classification offers a more ecologically informative approach, while concurrently simplifying taxonomic complexity. The explanatory power of the FG community structure by environmental factors slightly surpassed that of taxonomic structure, with Chl_a exhibiting the strongest correlation in both cases (0.631 and 0.582, respectively), followed by total phosphorus (0.363 and 0.362) and pH (0.389 and 0.246). Latitude, representing climatic geography, also made a significant contribution to explanatory rates (0.385 and 0.268). Notably, WIN_S_Max, DO, and total nitrogen did not significantly impact taxonomic composition (*p* > 0.05) but did exert a significant effect on FG composition (*p* < 0.05), underscoring the stronger correlation between FG structure and the environment. Notably, total nitrogen and ammonia nitrogen made a comparatively small contributions to community differences than total phosphorus content. This emphasized a preference for phosphorus limitation in the sampled lakes of North China. The strong correlations between EC, total dissolved solids, and SAL (Figure 5e) were moderated by RDA, highlighting that EC was a primary factor with stronger and more significant (*p* < 0.05) correlations explaining the community structure. Concerning other environmental factors related to climatic geography, all had moderate influences, except for atmospheric pressure, which did not correlate with differences in planktonic algal communities.

**FIGURE 6:**
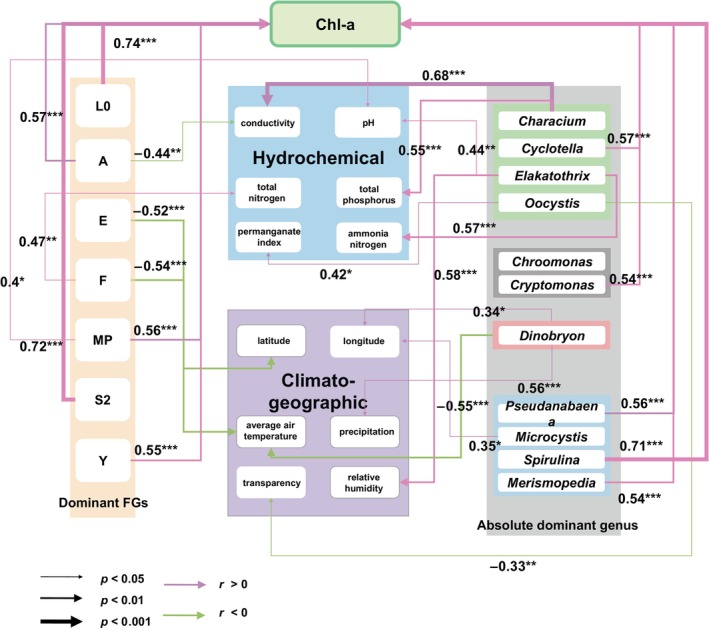
Spearman's rank correlations between environmental drivers, dominant FGs, absolutely dominant genera, and Chl_a content (2‐column fitting image). Dominant FGs: FGs comprising more than 10% of the total are deemed dominant FGs; Absolutely dominant genera: genera that Y_i_ > 0.1; Chl‐a: chlorophyll‐a concentration.

Given the extensive study area and prolonged sampling duration, the task of identifying key environmental drivers posed inherent challenges. To address this, we conducted additional Spearman's rank correlation coefficient analyses (refer to Figure 6, Figure S5 for details). The findings underscored the significant positive correlation between Chl_a content and both dominant FGs and absolutely dominant genera. Spirulina exhibited the highest correlation coefficient with Chl_a content, registering at 0.71, while other genera displayed correlation coefficients exceeding 0.5. Remarkably, the dominant FG at L0 recorded the highest correlation coefficient at 0.74, surpassing even that of the absolutely dominant genera. The correlation coefficients for other dominant FGs exhibiting significant correlations with Chl_a were marginally larger than those for the absolutely dominant genera.

In conclusion, we found that environmental factors had a greater influence on the structure of phytoplankton FGs than on taxa. Significantly, in this analysis, climatic–geographic factors exhibited more intricate correlation with community composition compared to hydrochemical factors. The influence of climatic–geographic factors on communities will reflect differences with the study area and the study scale (Winder and Sommer 2012; Lehman 2000). This discrepancy shows that there is potential for variability in results stemming from different scopes and analytical methodologies. Nevertheless, a consistent trend emerged in our study, indicating that the escalating influence of climatic and geographic factors may increase as the spatial scope expands.

### 3.3 East China Samples and Validation of Conclusions

**FIGURE 7:**
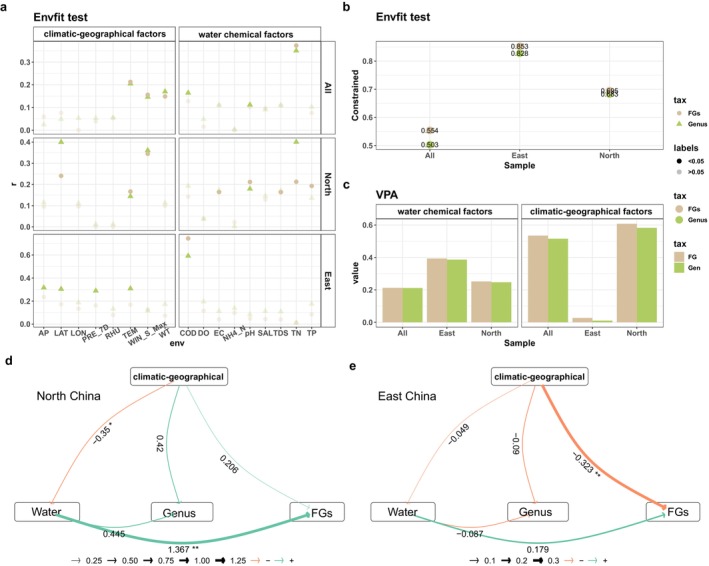
Comparison of sample sites in North China and East China. (a) Effects of various environmental factors on the phytoplankton community structure, (b) total explanation of the phytoplankton community structure by all environmental factors, (c) two types of environmental factors, (d) separate structural equation model of North China, and (e) separate structural equation model of East China.

The first two ordering axes of the PCoA explained 11.93% and 9.59% of the differences in the taxonomic structure and 15.54% and 11.32% of the differences in the FG structure, respectively (Figure S7). Similarly, the first two ordering axes of the RDA explained 21.52% and 11.25% of the differences in the taxonomic structure and 26.42% and 20.37% of the differences in the FG structure, respectively (Figure S8). Both methods demonstrated excellent capabilities to elucidate FG structures. Furthermore, the Envfit test demonstrated that the majority of environmental factors exerted a greater influence on the FG structure than on the taxonomic structure, and that the combined effects of these factors were more pronounced in FG than on taxa across all regions (Figure 7a–c). The results of the structural equation modeling revealed a significant impact of climatic–geographic and water chemical factors on the community structure. Additionally, there was a notable effect of climatic–geographic factors on water chemical factors (Figure 7d,e).

## 4 Discussion

### 4.1 FG Is an Ecological Simplification of Phytoplankton Taxonomic Composition

There were differences in the results of FG and taxonomic composition. This discrepancy arises from the classification of FGs, primarily determined by ecological functions, where genera with comparable habitats and ecological statuses are grouped together, resulting in a streamlined number of functional groupings (Reynolds 1980; Salmaso and Padisák 2007; Kruk et al. 2010).

Drawing insights from the results presented in Figures 2 and 3, it was evident that the community structures resulting from the two classification methods exhibit high degrees of similarity, with the FG classification proving to be more comprehensive. This classification method, while preserving all pertinent information, effectively streamlines details that bear minimal or negligible impacts on the outcomes. Essentially, the FG classification enhances the interpretability of results by prioritizing significant ecological patterns while maintaining a comprehensive overview of community structures.

The application of FG classification methods not only facilitates a simplified description of the community structure, but also offers a number of advantages in describing community diversity. While the coordinate axis absolute values of Richness and Shannon's index FG structure and taxonomic composition were different, their relative values were similar. This suggested that the overall trend of the community diversity index will not change, despite the slight differences in values when using FGs for classification rather than taxa. That is, communities with high diversity will not be incorrectly classified as low diversity, and vice versa. This ensures the accuracy of the description of community diversity.

Contemporary researchers in related fields are increasingly utilizing of FGs as a powerful tool to dissect phytoplankton communities, obtaining valuable insights into aquatic ecosystems (Estifanos, Gebre‐Meskel, and Hailu 2022; Wu et al. 2023; Yan et al. 2023). In this investigation, we employed the FG classification approach to illustrate this concept. Our findings revealed the striking similarity between phytoplankton FGs and their respective populations concerning the taxonomic structure and diversity composition. Also, in the FG structure PCoA, the distributions of repeated sampling points within groups were less dispersed than in the taxonomic composition, while the distributions of inter‐group samples were more dispersed (Figure 4), indicating that FG structure divisions are more conducive to informative analysis compared to taxonomic structure divisions. Collectively, our analysis indicated that the division of algal communities is not manifested in distinct characteristics based on diverse dividing principles. Instead, it revealed that there are comparable structural traits between algal community compositions and FGs, with the latter being more generalized compared to taxonomy. This approach is also less prone to be biased by inconsistencies in species identification (Znachor et al. 2020).

### 4.2 FG Structure Contains More Information on Ecological Features Than Taxonomic Composition

The results of the RDA, Mantel test (Figure 5), and correlation analyses (Figure 6) were consistent, and all showed that environmental factors had a stronger effect on the FG structure than taxonomic composition. This suggested that the FG structure contains more information on community ecological characteristics despite having fewer distinct units than taxonomic composition.

In ecological research, subtle disparities in morphological characteristics between organisms often have minimal significance in determining their ecological status. Therefore, emphasis should instead be placed on comprehending variations in ecological functions (Dong et al. 2013). The classification of FGs adheres to the ecological niche principle, relying on life habits and survival strategies as the foundation for categorization (Loreau et al. 2001). This approach effectively mitigates the influence of morphological and genomic differences while underscoring the importance of ecological functions. Furthermore, consolidating planktonic algae sharing similar habitats into FG simplifies complexity, thereby facilitating subsequent analyses (Borics et al. 2007; Kruk et al. 2010). For instance, here, 18 FGs encompassed 30 genera (top 30 genera shared in 11 lakes), representing 5 phyla (Figure 2d). The ecological focus of the grouping can be illustrated by looking at *Pseudanabaena* and *Navicula*, the two dominant genera in BYD Lake and FHEK Lake, which were classified into the same MP FG despite belonging to distinct phyla (Table S6).

Using FG classifications provides broader categorizations than using individual genera, while also providing more information than phylum level classifications. FG classifications take into account the ecological adaptations of planktonic algae, which makes it more suitable for ecological studies than relying solely on genomic differences. Because FG classifications will always be fewer than the number of genera given the same number of total cells (Becker et al. 2010), information redundancy will be reduced. Assigning some species with lower abundances to a disadvantaged FG can also help to avoid the “double‐zero problem” in ecological analyses (Borcard, Gillet, and Legendre 2011).

As FGs are somewhat contingent on the environment in which a community resides, they better reflect the similarties among environments (Mutshinda et al. 2016). This was evidenced by the reduction in β‐diversity relative to taxonomic composition (Figure S7d). These factors diminished the impact of the environment on FG differences, thereby making the effect of specific environmental factors on taxonomic composition more pronounced in East China (Figure 7a). However, the analyses conducted for each region indicated that environmental factors had a greater overall effect on explaining the structure of the phytoplankton FG (Figure 7b). This finding was in accordance with the conclusions presented in the preceding section. The VPAs of environmental factors by category also showed a more pronounced overall impact on FG composition for both climatic–geographical factors and water chemical factors (Figure 7c). This provided further evidence of the broad applicability of the findings. The definition of phytoplankton FGs reduces the significance of subtle morphological differences and completely invisible coding differences in the genome, shifting the research focus toward ecological adaptations (Salmaso, Naselli‐Flores, and Padisak 2015). Since species within the same FG tend to exhibit similar ecological traits, this classification provides more ecological information with fewer taxonomic units.

### 4.3 Climatic–Geographic Factors Exert Indirect Effects on Phytoplankton Communities Through Water Chemistry Factors

The role of climatic and geographical factors in shaping biological communities is well established. Our study, with an expanded geographic scope and an extended sampling period, revealed a strong impact of these factors on taxonomic construction. Although their influence did not surpass the individual explanatory power of nutrient effects on community structure, their effect was no longer negligible at this scale. For example, WIN_S_Max held a notable position on both axes for both taxonomic and FG classifications, indicating that the algal community structure is susceptible to wind and wave perturbations, which may be related to the shallow nature of the sampled lakes (Zhou et al. 2015). Furthermore, it is expected that climatic–geographic factors will be even more influential at wider geographical scopes and over longer sampling periods (Birk et al. 2020; Farrell et al. 2020). Notably, the Mantel test, conducted to visualize the effects of environmental factors on differences in algal community structure, indicated that a majority of climatic and geographic factors had significant effects on both taxonomic and FG community structures (Figure 5e).

In comparison to North China, East China is characterized by a greater abundance of rainfall and superior water network connectivity (Li et al. 2019; Zhao, Liu, and Luo 2020). Consequently, the degree of similarity in the physicochemical properties of water bodies between lakes is higher in East China (Qin et al. 2023). Additionally, due to the similarity among environments, the level of variation in the community structure (i.e., β‐diversity) of East China was lower than in North China (Figure S7d).

It is noteworthy that the impacts of climatic–geographical factors on the community structure were surprisingly limited in eastern China (Figure 7c). While this can be partially attributed to the region's more consistent climate, the underlying causes warrant further investigation. To this end, PLS_SEM was developed for the sample sites in each of the two regions (Figure 7d,e). The results revealed that climatic–geographical factors, in addition to directly influencing changes in phytoplankton communities, also exerted a pronounced impact on water chemical factors, particularly in North China (Figure 7d). This may have been due to the broader geographic scope of the sampling sites in North China and the diverse climatic patterns, which can exert intricate effects on water quality (Wang, Li, and Li 2018). Furthermore, in the majority of cases, environmental factors exerted a greater influence on FG composition than on taxonomic composition. While climatic–geographical factors in North China exerted a greater direct influence on taxonomic composition than on FG composition, the combination of their direct and indirect impacts had a greater impact on FG composition (Figure 7d).

While the direct impacts of geographical conditions on the phytoplankton community structure may be difficult to discern, their indirect effects should not be overlooked (Domis et al. 2013). In contrast to terrestrial ecosystems, freshwater ecosystems—particularly lake ecosystems—are relatively closed. In open terrestrial ecosystems, because individual animals can move freely through environments, communities of organisms are primarily shaped by environmental heterogeneity and interspecific interactions (Stein, Gerstner, and Kreft 2014). In contrast, in more closed lake ecosystems, phytoplankton movement is limited, and the community structure is more heavily regulated by the combined effects of nutrient availability and the environmental conditions of the water body in which they live (Stomp et al. 2011). The chemical conditions of lake water are largely influenced by the climate. For example, one of our sample sites, YCYH lake, a salt lake in Yuncheng, Shanxi, China, experiences hot and arid conditions during the summer months. This results in significant lake water evaporation, which in turn causes SAL and nutrient salt concentrations to rise considerably (Li et al. 2016). At this time, the lake's phytoplankton community undergoes a major shift, becoming dominated by salt‐tolerant species (Gao et al. 2021).

## 5 Conclusions

1. A comprehensive survey across 11 lakes or reservoirs revealed the presence of 81 phytoplankton genera spanning 7 phyla. The FG index was applied to facilitate the categorization of phytoplankton into 30 distinct groups. The number of FGs was less than the number of genera, yet the diversity of the phytoplankton community was well represented.
2. Significant parallels were identified between phytoplankton populations and FG structures, demonstrating coherence in structural composition and responsiveness to environmental drivers. The application of FG classification emerged as a valuable tool, offering a streamlined approach without losing essential details about community structure.
3. Both climactic and geographic of environmental drivers had greater influences on the structure of FGs than on taxonomic groups. This indicated that the FG classification method captures more information about the ecological characteristics of the phytoplankton community.
4. The direct effect of climatic–geographical factors on the phytoplankton community structure was lower than that of water chemistry, but the indirect effect was stronger. At larger study scales, climatic–geographical factors can influence phytoplankton communities through their influence on the physicochemical properties of the water.

## Author Contributions

**Wei Wang:** data curation (equal), investigation (equal), visualization (equal), writing – original draft (equal), writing – review and editing (equal). **Hanjie Huang:** investigation (equal). **Zhongshi He:** methodology (equal), software (equal), visualization (equal), writing – original draft (equal), writing – review and editing (equal). **Guotao Zhang:** investigation (equal). **Junping Lv:** methodology (equal), supervision (equal). **Qi Liu:** data curation (equal), resources (equal). **Fangru Nan:** data curation (equal), resources (equal). **Xudong Liu:** data curation (equal), resources (equal). **Shulian Xie:** conceptualization (equal), project administration (equal), supervision (equal). **Jia Feng:** conceptualization (equal), funding acquisition (equal), project administration (equal), supervision (equal), writing – review and editing (equal).

## Conflicts of Interest

The authors declare no conflicts of interest.

## Supporting information


**Data S1.** **Figure S1.** Environmental factors.
**Figure S2.** Comparison of the top five genera and functional groups by lake share.
**Figure S3.** Comparison of the dominant genera and functional groups.
**Figure S4.** Correlation analysis of taxonomy constructures: (a) FGs, (b) environmental factors, and relative abundance of phytoplankton in lakes.
**Figure S5.** Comparison of correlation between community structure and environmental factors. (a) Absolute abundance of genera, (b) relative abundance of genera, (c) absolute abundance of FGs, and (d) relative abundance of FGs.
**Figure S6.** Phytoplankton functional group and taxon correlation in North China.
**Figure S7.** Phytoplankton community structure in East China. (a) Principal coordinates analysis (PCoA) based on taxonomy composition and (b) PCoA based on the FG structure. (c) Phytoplankton functional group and taxon correlation in East China and (d) beta diversity of two areas.
**Figure S8.** Dynamic drivers of the phytoplankton community structure in eastern China. (a) RDA of taxonomy composition, (b) RDA of FGs, and (c) Mantel test.
**Table S1.** Sample information.
**Table S2.** Sample environmental factors.
**Table S3.** Taxonomy composition.
**Table S4.** FG structure.
**Table S5.** RDA ordering axis.
**Table S6.** Relationships between taxa and functional groups.
**Note S1.** Details for collection and analysis of biological samples.
**Note S2.** Taxonomy and FG composition of each lake.
**Note S3.** Correlation between phytoplankton and environmental factors.

## Data Availability

The authors confirm that the data supporting the findings of this study are available within the article and its appendix.

## References

[ece370656-bib-0001] Becker, V. , L. Caputo , J. Ordóñez , et al. 2010. “Driving Factors of the Phytoplankton Functional Groups in a Deep Mediterranean Reservoir.” Water Research 44, no. 11: 3345–3354. 20398914 10.1016/j.watres.2010.03.018

[ece370656-bib-0002] Birk, S. , D. Chapman , L. Carvalho , et al. 2020. “Impacts of Multiple Stressors on Freshwater Biota Across Spatial Scales and Ecosystems.” Nature Ecology & Evolution 4, no. 8: 1060–1068. 10.1038/s41559-020-1216-4. 32541802

[ece370656-bib-0003] Borcard, D. , F. Gillet , and P. Legendre . 2011. Numerical Ecology With R. New York: Springer.

[ece370656-bib-0004] Borics, G. , G. Várbíró , I. Grigorszky , E. Krasznai , S. Szabó , and K. T. Kiss . 2007. “A New Evaluation Technique of Potamo‐Plankton for the Assessment of the Ecological Status of Rivers.” Large Rivers 17: 466–486. 10.1127/lr/17/2007/466.

[ece370656-bib-0005] Chen, Q. , Q. H. Li , X. Y. Ma , M. J. Xiong , Y. He , and M. S. Han . 2019. “Comparison of Functional Groups of Phytoplankton in FG, MFG, and MBFG: Taking Three Reservoirs as an Example in Guizhou Plateau.” HuanJjing Kexue 40, no. 9: 4061–4071. 10.13227/j.hjkx.201901192. 31854869

[ece370656-bib-0007] Devlin, M. J. , M. Breckels , C. A. Graves , et al. 2019. “Seasonal and Temporal Drivers Influencing Phytoplankton Community in Kuwait Marine Waters: Documenting a Changing Landscape in the Gulf.” Frontiers in Marine Science 6: 141. 10.3389/fmars.2019.00141.

[ece370656-bib-0008] Domis, D. S. , N. Lisette , J. J. Elser , et al. 2013. “Plankton Dynamics Under Different Climatic Conditions in Space and Time.” Freshwater Biology 58, no. 3: 463–482. 10.1111/fwb.12053.

[ece370656-bib-0009] Dong, J. , Y. Li , G. Li , Y. Li , Y. Liu , and L. Song . 2013. “Seasonal Dynamics Characteristics and Affecting Physical Factors of Phytoplankton Functional Groups in Dongjiang River.” Acta Hydrobiologica Sinica 37, no. 5: 836–843. 10.7541/2013.107.

[ece370656-bib-0010] Dutkiewicz, S. , J. J. Morris , M. J. Follows , et al. 2015. “Impact of Ocean Acidification on the Structure of Future Phytoplankton Communities.” Nature Climate Change 5, no. 11: 1002–1006. 10.1038/nclimate2722.

[ece370656-bib-0011] Estifanos, G. , D. Gebre‐Meskel , and T. Hailu . 2022. “Water Quality Parameters Affect Dynamics of Phytoplankton Functional Groups in Lake Hawassa, Ethiopia.” Limnologica 94: 125968. 10.1016/j.limno.2022.125968.

[ece370656-bib-0012] Fang, K. , F. Nan , J. Feng , et al. 2022. “Sheathia Yunnanensis, a New Species of Freshwater Red Alga (Rhodophyta: Batrachospermales) From Yunnan, China.” Nordic Journal of Botany 2022, no. 5: e03476. 10.1111/njb.03476.

[ece370656-bib-0013] Farrell, K. , N. Ward , A. Krinos , et al. 2020. “Ecosystem‐Scale Nutrient Cycling Responses to Increasing Air Temperatures Vary With Lake Trophic State.” Ecological Modelling 430: 109134. 10.1016/j.ecolmodel.2020.109134.

[ece370656-bib-0015] Gao, F. , F. Nan , J. Feng , et al. 2021. “Transcriptome Profile of *Dunaliella salina* in Yuncheng Salt Lake Reveals Salt‐Stress‐Related Genes Under Different Salinity Stresses.” Journal of Oceanology and Limnology 39, no. 6: 2336–2362. 10.1007/s00343-021-0164-4.

[ece370656-bib-0016] Habib, O. , R. Tippett , and K. Murphy . 1997. “Seasonal Changes in Phytoplankton Community Structure in Relation to Physico‐Chemical Factors in Loch Lomond, Scotland.” Hydrobiologia 350: 63–79. 10.1023/A:1003037012226.

[ece370656-bib-0017] He, Z. , Y. Chen , D. Huo , et al. 2023. “Combined Methods Elucidate the Multi‐Organ Toxicity of Cylindrospermopsin (CYN) on *Daphnia magna* .” Environmental Pollution 324: 121250. 10.1016/j.envpol.2023.121250. 36813104

[ece370656-bib-0018] Hu Ren, L. A. N. , X. I. A. O. L. Yuqian , and H. A. N. Boping . 2015. “The Concepts, Classification and Application of Freshwater Phytoplankton Functional Groups.” Journal of Lake Science 27, no. 1: 11–23.

[ece370656-bib-0019] Huang, G. , Q. Li , C. Chen , et al. 2015. “Phytoplankton Functional Groups and Their Spatial and Temporal Distribution Characteristics in Hongfeng Reservoir, Guizhou Province.” Acta Ecologica Sinica 35, no. 17: 5573–5584. 10.13671/j.hjkxxb.2014.0747.

[ece370656-bib-0020] Kruk, C. , V. L. Huszar , E. T. Peeters , et al. 2010. “A Morphological Classification Capturing Functional Variation in Phytoplankton.” Freshwater Biology 55, no. 3: 614–627. 10.1111/j.1365-2427.2009.02298.x.

[ece370656-bib-0021] Lehman, P. W. 2000. “The Influence of Climate on Phytoplankton Community Biomass in San Francisco Bay Estuary.” Limnology and Oceanography 45, no. 3: 580–590. 10.4319/lo.2000.45.3.0580.

[ece370656-bib-0022] Li, C. , T. Liu , S. Xu , X. Gao , and Y. Wang . 2016. “Groundwater Salinization in Shallow Aquifers Adjacent to a Low‐Altitude Inland Salt Lake: A Case Study at Yuncheng Basin, Northern China.” Environmental Earth Sciences 75: 1–14. 10.1007/s12665-016-5260-y.

[ece370656-bib-0024] Li, Y. , Q. Zhang , Y. Cai , et al. 2019. “Hydrodynamic Investigation of Surface Hydrological Connectivity and Its Effects on the Water Quality of Seasonal Lakes: Insights From a Complex Floodplain Setting (Poyang Lake, China).” Science of the Total Environment 660: 245–259. 10.1016/j.scitotenv.2019.01.015. 30640093

[ece370656-bib-0025] Liu, Q. , F. Chang , P. Xie , et al. 2023. “Microbiota Assembly Patterns and Diversity of Nine Plateau Lakes in Yunnan, Southwestern *China* .” Chemosphere 314: 137700. 10.1016/j.chemosphere.2022.137700. 36587916

[ece370656-bib-0026] Liu, W. , D. W. Au , D. M. Anderson , P. K. Lam , and R. S. Wu . 2007. “Effects of Nutrients, Salinity, pH and Light: Dark Cycle on the Production of Reactive Oxygen Species in the Alga Chattonella Marina.” Journal of Experimental Marine Biology and Ecology 346, no. 1–2: 76–86.

[ece370656-bib-0027] Loreau, M. , S. Naeem , P. Inchausti , et al. 2001. “Biodiversity and Ecosystem Functioning: Current Knowledge and Future Challenges.” Science 294, no. 5543: 804–808. 10.1126/science.1064088. 11679658

[ece370656-bib-0029] Ma, Z. , W. Ma , X. Li , Z. Li , M. Pan , and C. Ding . 2022. “Succession Characteristics of Phytoplankton Functional Groups and Water Quality Responsiveness Evaluation in an Artificial Constructed Wetland‐Reservoir Ecosystem.” Environmental Pollutants and Bioavailability 34, no. 1: 202–214. 10.1080/26395940.2022.2077836.

[ece370656-bib-0030] McNaughton, S. J. 1967. “Relationships Among Functional Properties of Californian Grassland.” Nature 216, no. 5111: 168–169. 10.1038/2171163a0.

[ece370656-bib-0031] Meng, X. , W. Zhang , and B. Shan . 2021. “Evaluating the Biotoxicity of Surface Water in a Grassy Lake in North China.” Journal of Environmental Sciences 102: 316–325. 10.1016/j.jes.2020.09.028. 33637257

[ece370656-bib-0032] Mousing, E. A. , M. Ellegaard , and K. Richardson . 2014. “Global Patterns in Phytoplankton Community Size Structure—Evidence for a Direct Temperature Effect.” Marine Ecology Progress Series 497: 25–38. 10.3354/meps10583.

[ece370656-bib-0033] Mutshinda, C. M. , Z. V. Finkel , C. E. Widdicombe , and A. J. Irwin . 2016. “Ecological Equivalence of Species Within Phytoplankton Functional Groups.” Functional Ecology 30, no. 10: 1714–1722. 10.1111/1365-2435.12641.

[ece370656-bib-0034] Neri, F. , T. Romagnoli , S. Accoroni , et al. 2023. “Phytoplankton Communities in a Coastal and Offshore Stations of the Northern Adriatic Sea Approached by Network Analysis and Different Statistical Descriptors.” Estuarine, Coastal and Shelf Science 282: 108224. 10.1016/j.ecss.2023.108224.

[ece370656-bib-0035] Qin, B. , R. Wang , X. Yang , Q. Zhang , and J. Zheng . 2023. “Reconstruction and Trends of Total Phosphorus in Shallow Lakes in Eastern China in the Past Century.” Sustainability 15, no. 14: 10893. 10.3390/su151410893.

[ece370656-bib-0036] Reynolds, C. S. 1980. “Phytoplankton Assemblages and Their Periodicity in Stratifying Lake Systems.” Ecography 3, no. 3: 141–159.

[ece370656-bib-0037] Salmaso, N. , L. Naselli‐Flores , and J. Padisak . 2015. “Functional Classifications and Their Application in Phytoplankton Ecology.” Freshwater Biology 60, no. 4: 603–619. 10.1111/fwb.12520.

[ece370656-bib-0038] Salmaso, N. , and J. Padisák . 2007. “Morpho‐Functional Groups and Phytoplankton Development in Two Deep Lakes (Lake Garda, Italy and Lake Stechlin, Germany).” Hydrobiologia 578, no. 1: 97–112. 10.1007/s10750-006-0437-0.

[ece370656-bib-0039] Stein, A. , K. Gerstner , and H. Kreft . 2014. “Environmental Heterogeneity as a Universal Driver of Species Richness Across Taxa, Biomes and Spatial Scales.” Ecology Letters 17, no. 7: 866–880. 10.1111/ele.12277. 24751205

[ece370656-bib-0040] Stomp, M. , J. Huisman , G. G. Mittelbach , E. Litchman , and C. A. Klausmeier . 2011. “Large‐Scale Biodiversity Patterns in Freshwater Phytoplankton.” Ecology 92, no. 11: 2096–2107. 10.1890/10-1023.1. 22164834

[ece370656-bib-0041] Titilade, P. R. , and E. I. Olalekan . 2015. “The Importance of Marine Genomics to Life.” Journal of Ocean Research 3, no. 1: 1–13.

[ece370656-bib-0042] Wang, X. , Z. Li , and M. Li . 2018. “Impacts of Climate Change on Stream Flow and Water Quality in a Drinking Water Source Area, Northern China.” Environmental Earth Sciences 77: 1–14. 10.1007/s12665-018-7581-5.

[ece370656-bib-0043] Winder, M. , and U. Sommer . 2012. “Phytoplankton Response to a Changing Climate.” Hydrobiologia 698: 5–16. 10.1007/s10750-012-1149-2.

[ece370656-bib-0044] Wu, Z. , F. Wang , X. Wang , K. Li , and L. Zhang . 2023. “Water Quality Assessment Using Phytoplankton Functional Groups in the Middle‐Lower Changjiang River, China.” Limnologica 99: 126056. 10.1016/j.limno.2023.126056.

[ece370656-bib-0045] Xiang, R. , Q. Y. Li , Y. Yu , et al. 2017. “Functional Group Characteristics of Planktonic Diatoms and Their Relationship With Environmental Factors in the Ruxi River.” Huanjing Kexue 38, no. 8: 3290–3301. 10.13227/j.hjkx.201701111. 29964937

[ece370656-bib-0046] Xu, Y. , L. Wang , Q. Tang , L. Naselli‐Flores , E. Jeppesen , and B. P. Han . 2023. “The Relationship Between Phytoplankton Diversity and Ecosystem Functioning Changes With Disturbance Regimes in Tropical Reservoirs.” Ecosystems 26, no. 4: 752–767. 10.1007/s10021-022-00791-4.

[ece370656-bib-0047] Yan, G. , X. Yin , M. Huang , X. Wang , D. Huang , and D. Li . 2023. “Dynamics of Phytoplankton Functional Groups in River‐Connected Lakes and the Major Influencing Factors: A Case Study of Dongting Lake, China.” Ecological Indicators 149: 110177. 10.1016/j.ecolind.2023.110177.

[ece370656-bib-0048] Yang, J. , F. Wang , J. Lv , et al. 2019. “Responses of Freshwater Algal Cell Density to Hydrochemical Variables in an Urban Aquatic Ecosystem, Northern China.” Environmental Monitoring and Assessment 191: 1–16. 10.1007/s10661-018-7177-2. 30591969

[ece370656-bib-0049] Yang, W. , J. Zhu , K. Lu , L. Wan , and X. Mao . 2014. “The Establishment, Development and Application of Classification Approach of Freshwater Phytoplankton Based on the Functional Group: A Review.” Chinese Journal of Applied Ecology 25, no. 6: 1833–1840. 25223045

[ece370656-bib-0050] Yang, X. , L. Liu , X. Liu , S. Xie , J. Feng , and J. Lv . 2023. “The Responding Mechanism of Indigenous Bacteria in Municipal Wastewater Inoculated With Different Concentrations of Exogenous Microalgae.” Journal of Environmental Management 345: 118547. 10.1016/j.jenvman.2023.118547. 37433233

[ece370656-bib-0051] Zhang, M. , D. Straile , F. Chen , et al. 2018. “Dynamics and Drivers of Phytoplankton Richness and Composition Along Productivity Gradient.” Science of the Total Environment 625: 275–284. 10.1016/j.scitotenv.2017.12.288. 29289776

[ece370656-bib-0053] Zhao, L. , Y. Liu , and Y. Luo . 2020. “Assessing Hydrological Connectivity Mitigated by Reservoirs, Vegetation Cover, and Climate in Yan River Watershed on the Loess Plateau, China: The Network Approach.” Watermark 12, no. 6: 1742. 10.3390/w12061742.

[ece370656-bib-0054] Zhao, L. , T. Ouyang , L. Ji , et al. 2023. “Impounding Impacts of the Three Gorges Reservoir on Phytoplankton Function Groups and Its Relationship With Resource Use Efficiency.” Huan Jing Ke Xue 44, no. 2: 857–867. 10.13227/j.hjkx.202203240. 36775609

[ece370656-bib-0055] Zhou, J. , B. Qin , C. Casenave , et al. 2015. “Effects of Wind Wave Turbulence on the Phytoplankton Community Composition in Large, Shallow Lake Taihu.” Environmental Science and Pollution Research 22: 12737–12746. 10.1007/s11356-015-4535-2. 25913313

[ece370656-bib-0056] Znachor, P. , J. Nedoma , J. Hejzlar , et al. 2020. “Changing Environmental Conditions Underpin Long‐Term Patterns of Phytoplankton in a Freshwater Reservoir.” Science of the Total Environment 710: 135626. 31784170 10.1016/j.scitotenv.2019.135626

