# Supplementary material for: Unveiling Phytoplankton Diversity: Taxonomy, Functional Groups, and Environmental Drivers in North China Lakes
Source: Ecol Evol. 2024 Dec 3;14(12):e70656. doi: 10.1002/ece3.70656 (PMC11615088; doi:10.1002/ece3.70656)
Supplement: Supplementary file 1 — Data S1. Figure S1. Environmental factors. Figure S2. Comparison of the top five genera and functional groups by lake share. Figure S3. Comparison of the dominant genera and functional groups. Figure S4. Correlation analysis of taxonomy constructures: (a) FGs, (b) environmental factors, and relative abundance of phytoplankton in lakes. Figure S5. Comparison of correlation between community structure and environmental factors. (a) Absolute abundance of genera, (b) relative abundance of genera, (c) absolute abundance of FGs, and (d) relative abundance of FGs. Figure S6. Phytoplankton functional group and taxon correlation in North China. Figure S7. Phytoplankton community structure in East China. (a) Principal coordinates analysis (PCoA) based on taxonomy composition and (b) PCoA based on the FG structure. (c) Phytoplankton functional group and taxon correlation in East China and (d) beta diversity of two areas. Figure S8. Dynamic drivers of the phytoplankton community structure in eastern China. (a) RDA of taxonomy composition, (b) RDA of FGs, and (c) Mantel test. Table S1. Sample information. Table S2. Sample environmental factors. Table S3. Taxonomy composition. Table S4. FG structure. Table S5. RDA ordering axis. Table S6. Relationships between taxa and functional groups. Note S1. Details for collection and analysis of biological samples. Note S2. Taxonomy and FG composition of each lake. Note S3. Correlation between phytoplankton and environmental factors. [file ECE3-14-e70656-s001.docx]

**Unveiling Phytoplankton Diversity: Taxonomy, Functional Groups, and Environmental Drivers in North China Lakes**

Wei Wang^a^, Hanjie Huang^a^, Zhongshi He^b^, Guotao Zhang^a^, Junping Lv^a^, Qi Liu^a^, Fangru Nan^a^, Xudong Liu^a^, Yang Liu^a^, Shulian Xie^a^, Jia Feng^a, *^

**Fig. S1. Environmental factors**

**Fig. S2. Comparison of the top five genera and functional groups by lake share**

**Fig. S3. Comparison of the dominan genera and functional groups**

**Fig. S4. Correlation analysis of taxonomy constructures:** (a) FGs, (b) Environmental factors, and relative abundance of phytoplankton in lakes.

**Fig.S5. Comparison of Correlation between community structure and environmental factors.** (a) absolute abundance of genera, (b) relative abundance of genera, (c) absolute abundance of FGs, (b) relative abundance of FGs.

**Fig. S6. Phytoplankton functional group and taxon correlation in North China.**

**Fig. S7. Phytoplankton community structure in East China.** (a) Principal Coordinates Analysis (PCoA) based on taxonomy composition, and (b) PCoA based on FG structure. (c) Phytoplankton functional group and taxon correlation in East China, (d) Beta diversity of two areas.

**Fig. S8. Dynamic drivers of phytoplankton community structure in eastern China.** (a) RDA of taxonomy composition, (b) RDA of FGs, (c) Mantel test.

**Table S1 Sample information**

**Table S2 Sample environmental factors**

**Table S3 Taxonomy composition**

**Table S4 FG structure**

**Table S5 RDA ordering axis**

**Table S6 Relationships between taxa and functional groups**

**Note S1: Details for collection and analysis of biological samples.**

**Note S2: Taxonomy and FG composition of each lake.**

**Note S3: Correlation between phytoplankton and environmental factors.**


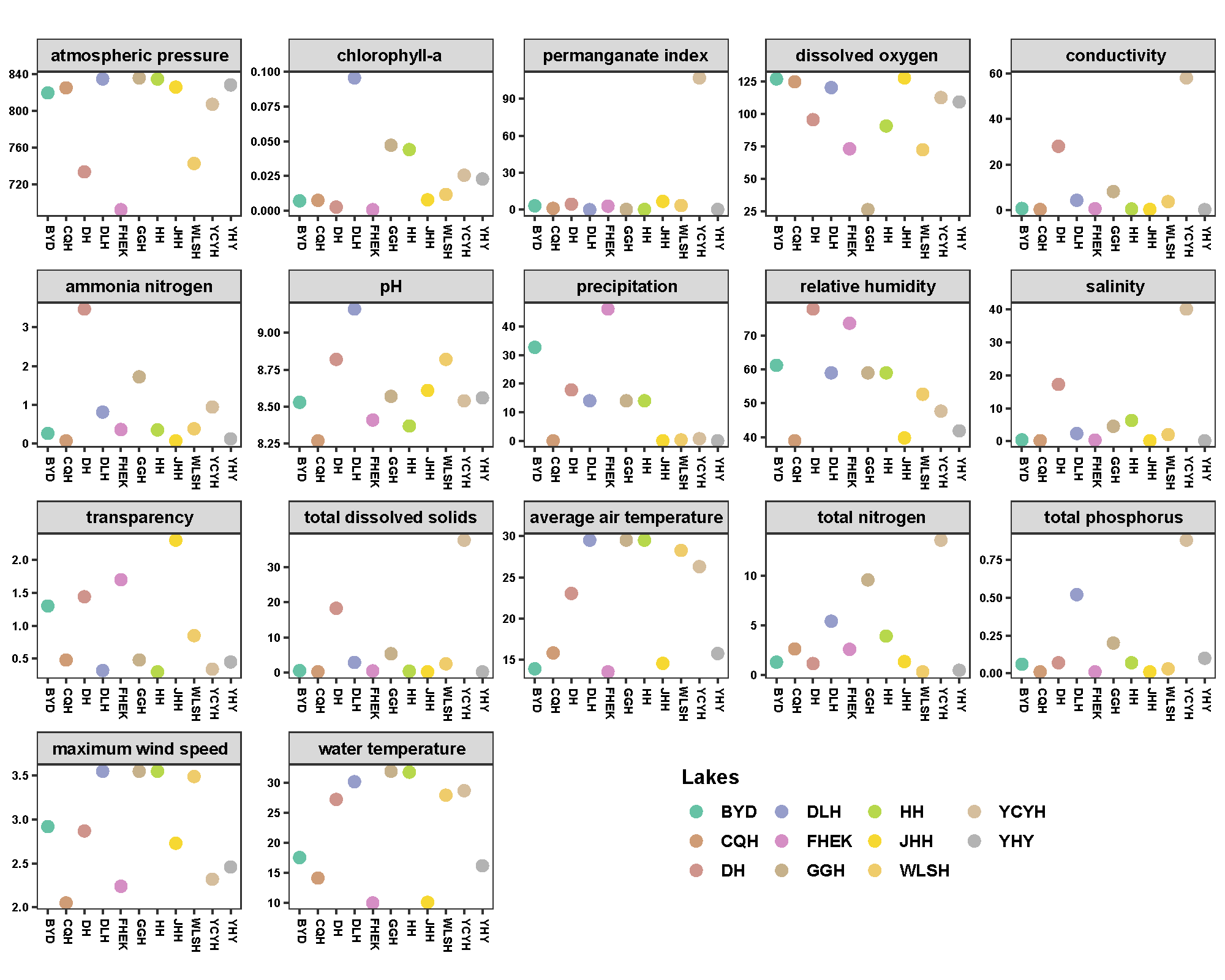


**Fig.S1 Environmental factors**


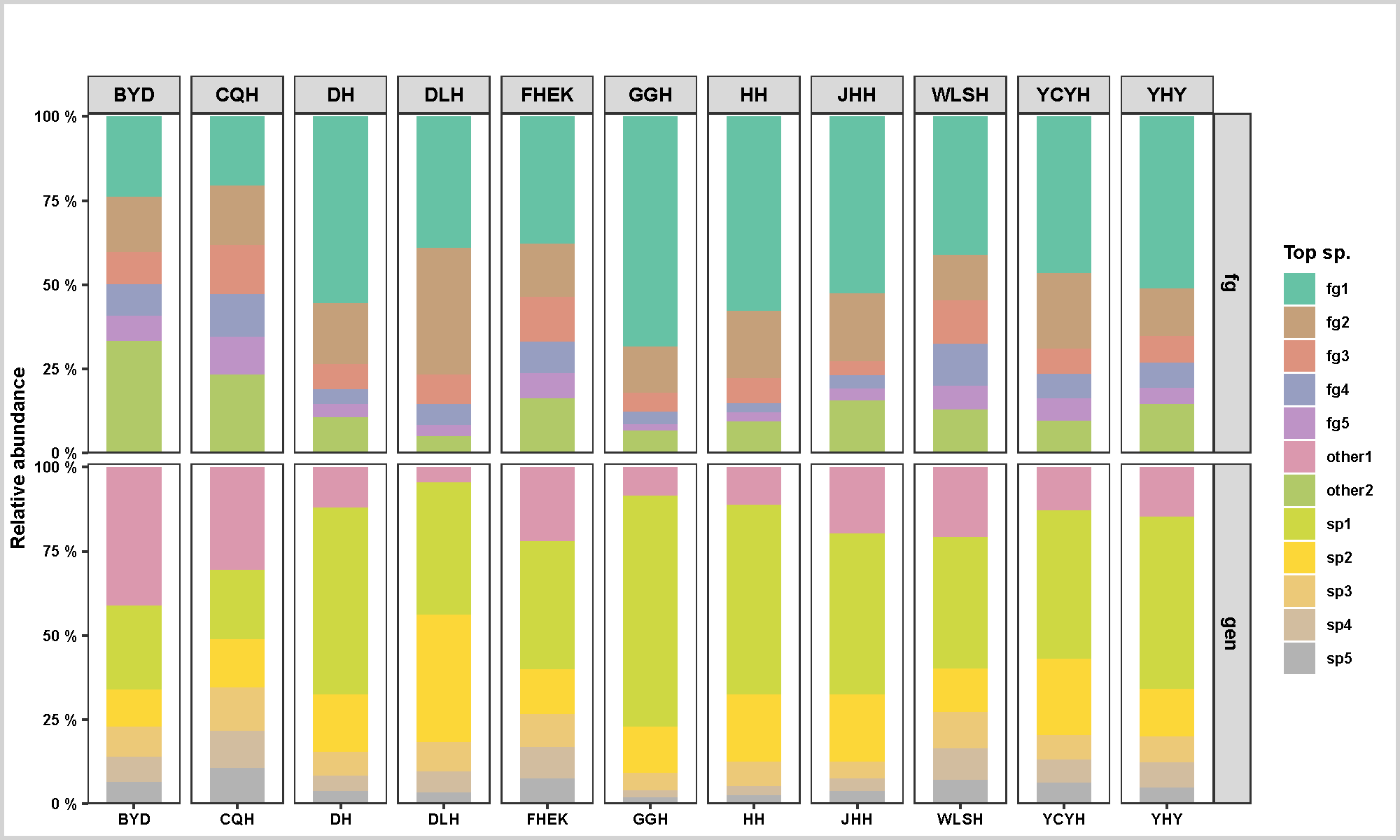


**Fig.S2 Comparison of the top five genera and functional groups by lake share**


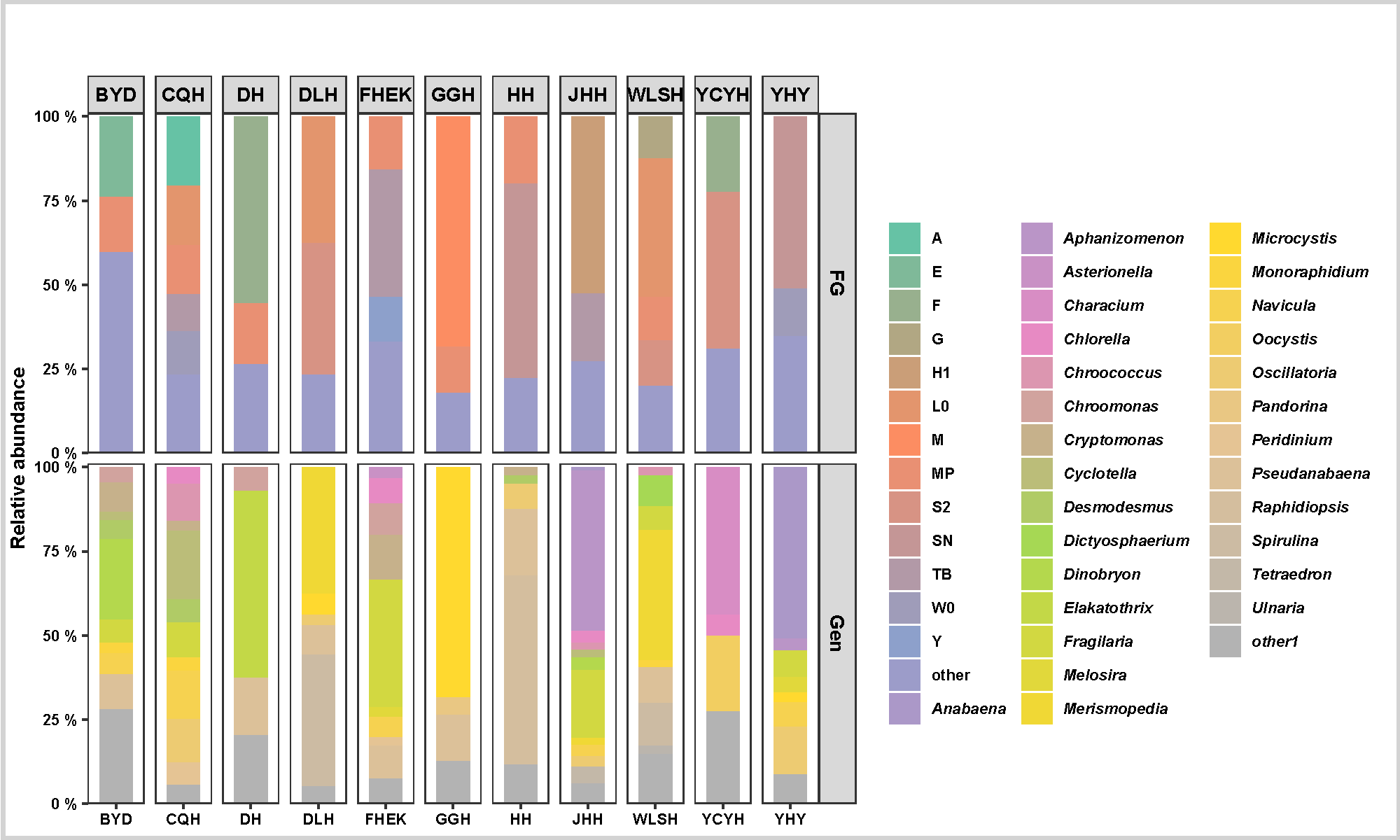


**Fig.S3 Comparison of the dominan genera and functional groups**

**
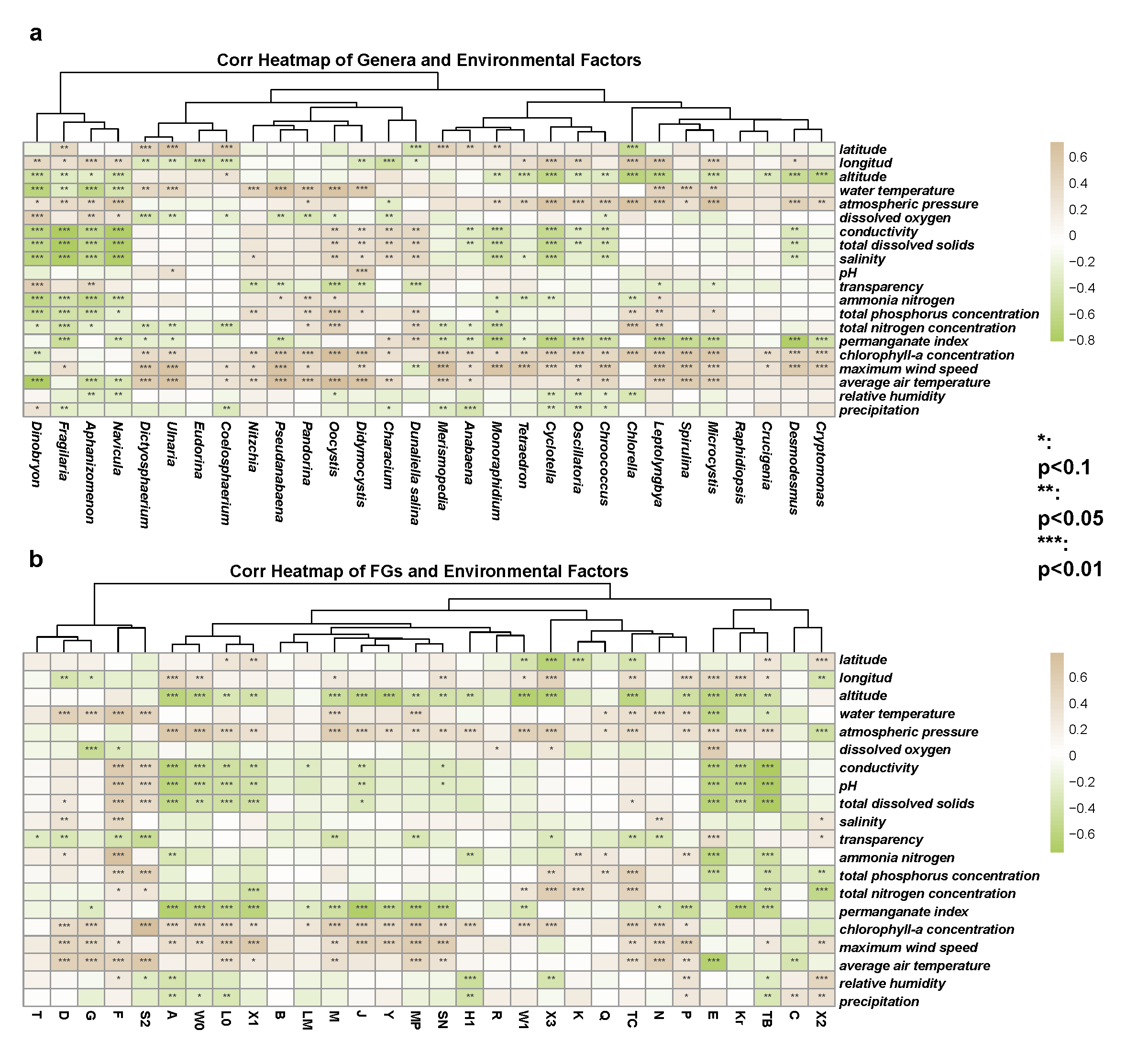
**

**Fig. S4. Correlation analysis of taxonomy constructures:** (a) FGs, (b) Environmental factors, and relative abundance of phytoplankton in lakes.

[2-column fitting image]

**
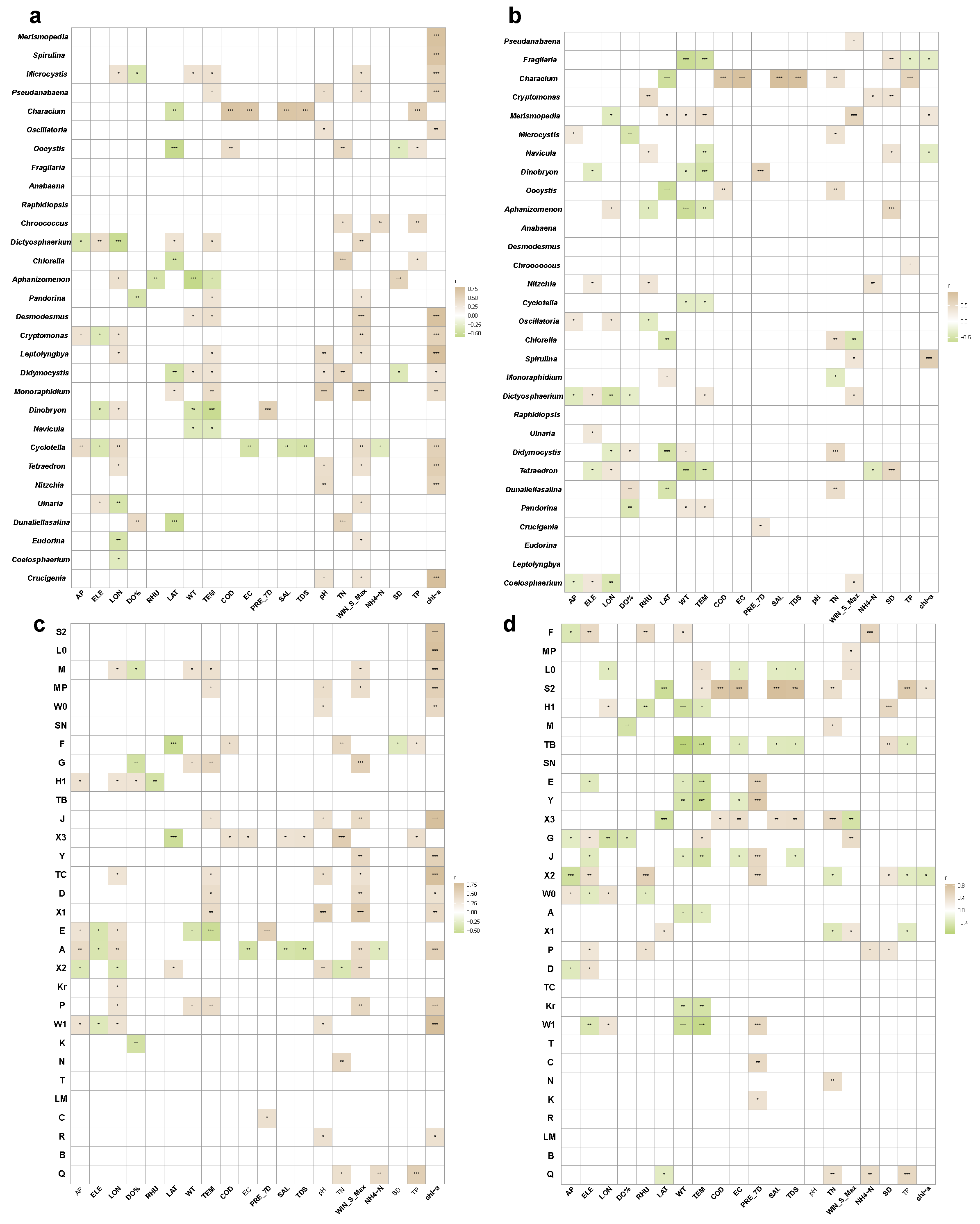
**

**Fig.S5 Comparison of Correlation between community structure and environmental factors. (a) absolute abundance of genera, (b) relative abundance of genera, (c) absolute abundance of FGs, (b) relative abundance of FGs.**

**
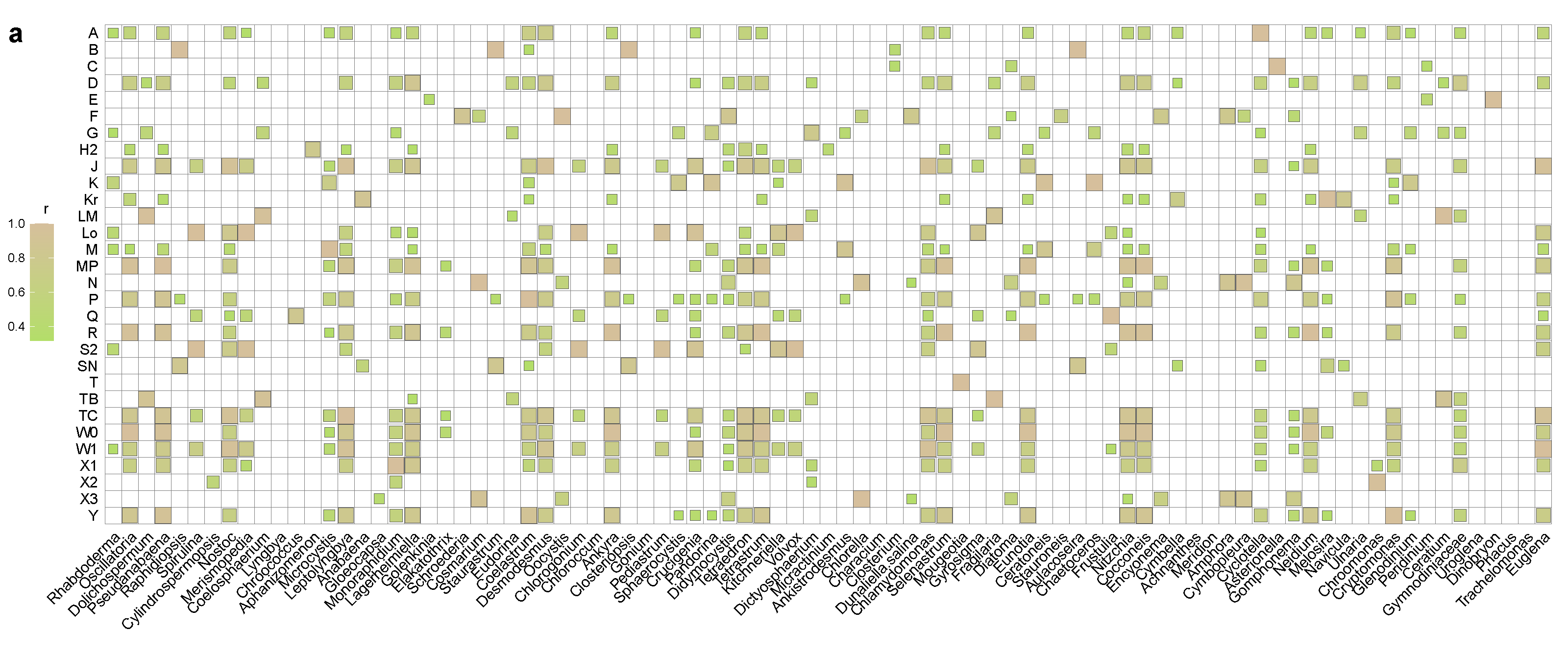
**

**Fig. S6. Phytoplankton functional group and taxon correlation in North China.**

**
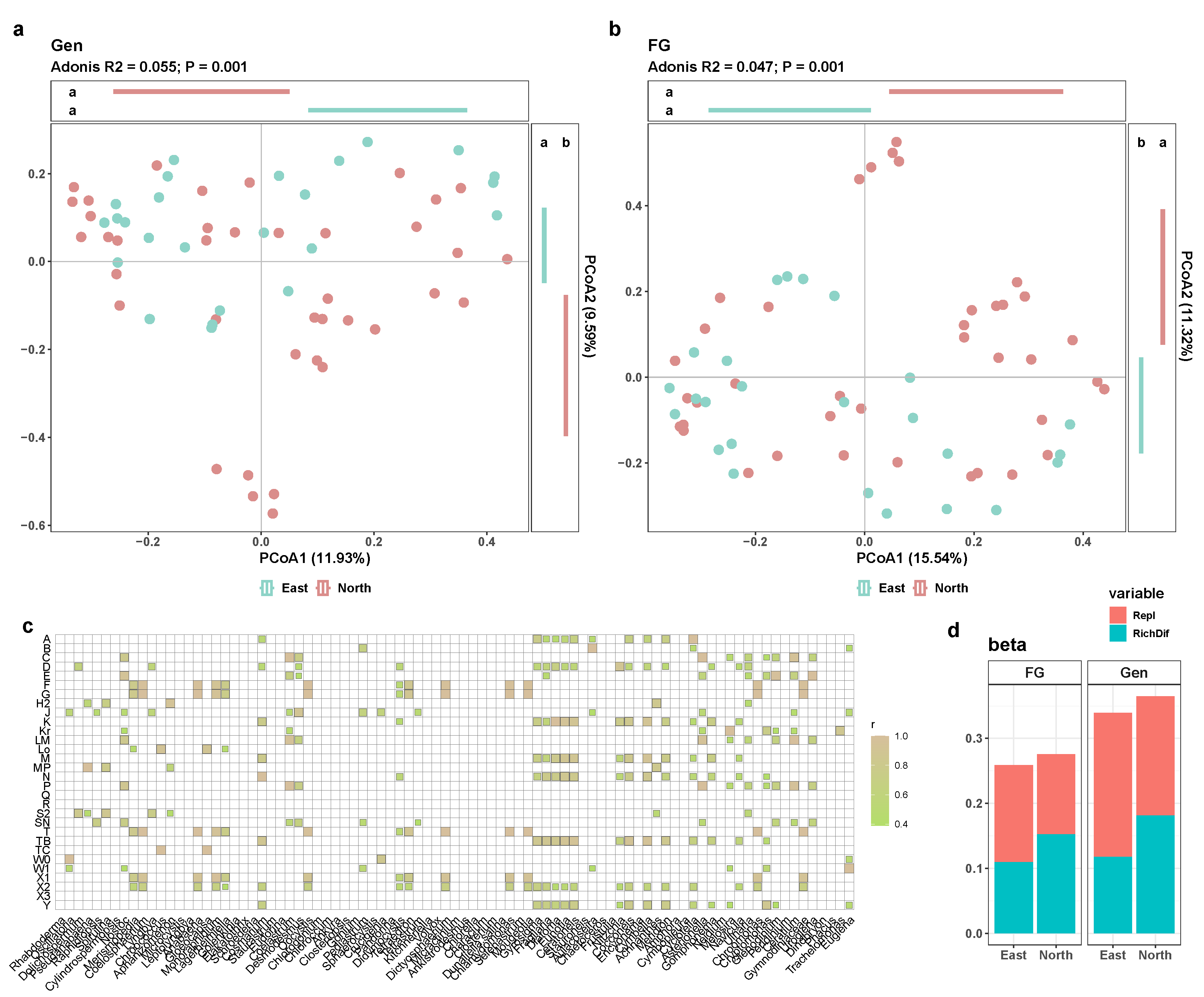
**

**Fig. S7. Phytoplankton community structure in East China.** (a) Principal Coordinates Analysis (PCoA) based on taxonomy composition, and (b) PCoA based on FG structure. (c) Phytoplankton functional group and taxon correlation in East China, (d) Beta diversity of two areas.

**
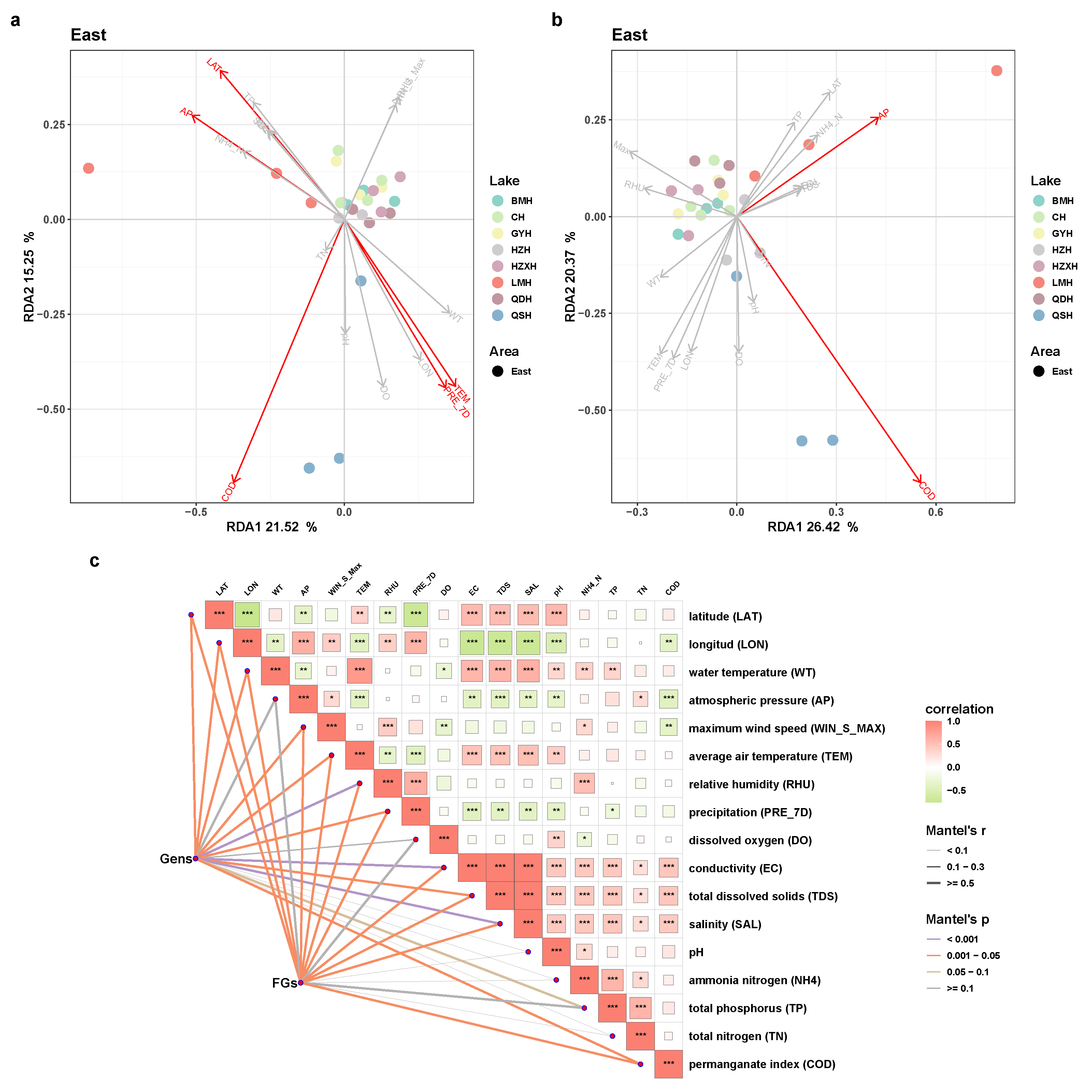
**

**Fig. S8. Dynamic drivers of phytoplankton community structure in eastern China.** (a) RDA of taxonomy composition, (b) RDA of FGs, (c) Mantel test.

**Table 1 Sample information**

| Abbreviations | City | Latitude and longitude |
| --- | --- | --- |
| CQH | Beijing City | 39.796389°N；116.080139°E |
| YHY | Beijing City | 39.997222°N；116.271528°E |
| JHH | Beijing City | 40.179815°N；117.301389°E |
| DLH | Tianjin City | 39.167039°N；117.474401°E |
| GGH | Tianjin City | 38.921014°N；117.516696°E |
| HH | Tianjin City | 39.153429°N；117.181087°E |
| DH | Inner Mongolia Autonomous Region | 40.593133°N；112.669639°E |
| WLSH | Inner Mongolia Autonomous Region | 40.836795°N；108.760124°E |
| BYD | Hebei Province | 38.930833°N；116.014206°E |
| FHEK | Shanxi Province | 37.981667°N；112.376944°E |
| YCYH | Shanxi Province | 34.971071°N；110.952619°E |
| LMH | Suqian City | 34.02903°N；118.2599°E |
| HZH | Sihong City | 33.25426°N；118.2562°E |
| BMH | Huaian City | 33.31249°N；119.058°E |
| GYH | Gaoyou City | 32.99683°N；119.4069°E |
| CH | Chaohu City | 31.4266°N；117.5503°E |
| QDH | Hangzhou City | 29.63558°N；119.0411°E |
| QSH | Hangzhou City | 30.2533°N；119.7596°E |
| HZXH | Hangzhou City | 30.24298°N；120.1276°E |

**Table S2 Sample environmental factors**

|  | WT(℃) | AP(Pa) | DO(%) | EC(-ms/cm) | TDS(g/L) | SAL(ppt) | pH | SD(m) | NH_4_^+^-N(mg/L) | TP(mg/L) | TN(mg/L) | CODMn | chl-a(mg/L) | WIN_S_Max(m/s) | TEM(℃) | RHU(%) | PRE_7D(mm) |
| --- | --- | --- | --- | --- | --- | --- | --- | --- | --- | --- | --- | --- | --- | --- | --- | --- | --- |
| CQH | 14.15 | 825.1 | 124.85 | 0.37 | 0.23 | 0.18 | 8.27 | 0.48 | 0.07 | 0.01 | 2.61 | 0.92 | 0.00739 | 2.05 | 15.84 | 38.94 | 0 |
| YHY | 16.2 | 828.25 | 109.35 | 0.29 | 0.19 | 0.14 | 8.56 | 0.45 | 0.12 | 0.1 | 0.45 | 0.04 | 0.02278 | 2.46 | 15.77 | 41.87 | 0 |
| JHH | 10.1 | 825.9 | 127.83 | 0.4 | 0.21 | 0.15 | 8.61 | 2.3 | 0.07 | 0.01 | 1.33 | 6.6 | 0.00775 | 2.73 | 14.58 | 39.77 | 0 |
| DLH | 30.2 | 834.8 | 120.4 | 4.4 | 2.86 | 2.33 | 9.16 | 0.32 | 0.81 | 0.52 | 5.41 | -0.14 | 0.0956 | 3.55 | 29.51 | 58.96 | 14 |
| GGH | 31.95 | 835.85 | 26.25 | 8.22 | 5.35 | 4.52 | 8.57 | 0.48 | 1.72 | 0.2 | 9.59 | 0.02 | 0.04709 | 3.55 | 29.51 | 58.96 | 14 |
| HH | 31.8 | 834.7 | 90.8 | 0.59 | 0.38 | 6.28 | 8.37 | 0.3 | 0.35 | 0.07 | 3.91 | 0.06 | 0.04394 | 3.55 | 29.51 | 58.96 | 14 |
| DH | 27.25 | 733.7 | 95.68 | 28.05 | 18.26 | 17.24 | 8.82 | 1.44 | 3.47 | 0.07 | 1.13 | 4.31 | 0.0025 | 2.87 | 23.05 | 77.76 | 17.8 |
| WLSH | 27.95 | 742.82 | 72.42 | 3.78 | 2.47 | 2 | 8.82 | 0.85 | 0.38 | 0.03 | 0.29 | 3.24 | 0.0116 | 3.49 | 28.27 | 52.65 | 0.3 |
| BYD | 17.56 | 819.64 | 127.11 | 0.81 | 0.53 | 0.4 | 8.53 | 1.3 | 0.26 | 0.06 | 1.27 | 2.98 | 0.00699 | 2.92 | 13.92 | 61.18 | 32.7 |
| FHEK | 10 | 692.6 | 73.2 | 0.72 | 0.47 | 0.36 | 8.41 | 1.7 | 0.36 | 0.01 | 2.57 | 2.67 | 0.00065 | 2.24 | 13.55 | 73.58 | 46.1 |
| YCYH | 28.71 | 807.2 | 112.7 | 57.98 | 37.63 | 40.08 | 8.54 | 0.34 | 0.94 | 0.88 | 13.61 | 106.99 | 0.0255 | 2.32 | 26.3 | 47.67 | 0.7 |

**Table S3 Taxonomy composition**

| **Genera** | **Abundance(cell/L)** | **Percentage** | **Genera** | **Abundance(cell/L)** | **percentage** |
| --- | --- | --- | --- | --- | --- |
| *Rhabdoderma* | 605000 | 0.0200% | *Micractinium* | 40000 | 0.0000% |
| *Oscillatoria* | 67000000 | 2.7600% | *Ankistrodesmus* | 100000 | 0.0000% |
| *Dolichospermum* | 1800000 | 0.0700% | *Chlorella* | 22495000 | 0.9300% |
| *Pseudanabaena* | 211073400 | 8.6900% | *Characium* | 94420000 | 3.8900% |
| *Raphidiopsis* | 31800000 | 1.3100% | *Closterium* | 100000 | 0.0000% |
| *Spirulina* | 624100000 | 25.7000% | *Dunaliella salina* | 5660000 | 0.2300% |
| *Cylindrospermopsis* | 420000 | 0.0200% | *Chlamydomonas* | 1606600 | 0.0700% |
| *Nostoc* | 2045000 | 0.0800% | *Selenastrum* | 500000 | 0.0200% |
| *Merismopedia* | 671120000 | 27.6300% | *Mougeotia* | 450000 | 0.0200% |
| *Coelosphaerium* | 5520000 | 0.2300% | *Gyrosigma* | 90000 | 0.0000% |
| *Chroococcus* | 29748400 | 1.2200% | *Fragilaria* | 36333400 | 1.5000% |
| *Aphanizomenon* | 19600000 | 0.8100% | *Diatoma* | 173400 | 0.0100% |
| *Microcystis* | 286830000 | 11.8100% | *Eunotia* | 500000 | 0.0200% |
| *Leptolyngbya* | 13550000 | 0.5600% | *Ceratoneis* | 180000 | 0.0100% |
| *Anabaena* | 33100000 | 1.3600% | *Stauroneis* | 20000 | 0.0000% |
| *Gloeocapsa* | 1800000 | 0.0700% | *Aulacoseira* | 80000 | 0.0000% |
| *Monoraphidium* | 10971600 | 0.4500% | *Chaetoceros* | 1880000 | 0.0800% |
| *Lagerheimiella* | 1940000 | 0.0800% | *Frustulia* | 65000 | 0.0000% |
| *Golenkinia* | 20000 | 0.0000% | *Nitzchia* | 7125000 | 0.2900% |
| *Elakatothrix* | 5180000 | 0.2100% | *Cocconeis* | 1645000 | 0.0700% |
| *Schroederia* | 1080000 | 0.0400% | *Encyonema* | 280000 | 0.0100% |
| *Cosmarium* | 1390000 | 0.0600% | *Cymbella* | 1915000 | 0.0800% |
| *Staurastrum* | 40000 | 0.0000% | *Amphora* | 60000 | 0.0000% |
| *Eudorina* | 5600000 | 0.2300% | *Cymbopleura* | 140000 | 0.0100% |
| *Coelastrum* | 4620000 | 0.1900% | *Cyclotella* | 7528400 | 0.3100% |
| *Desmodesmus* | 17175000 | 0.7100% | *Asterionella* | 206600 | 0.0100% |
| *Oocystis* | 53920000 | 2.2200% | *Gomphonema* | 505000 | 0.0200% |
| *Chlorogonium* | 220000 | 0.0100% | *Neidium* | 120000 | 0.0000% |
| *Chloroccum* | 960000 | 0.0400% | *Melosira* | 4955000 | 0.2000% |
| *Ankyra* | 425000 | 0.0200% | *Navicula* | 10165000 | 0.4200% |
| *Closteriopsis* | 20000 | 0.0000% | *Ulnaria* | 6685000 | 0.2800% |
| *Pediastrum* | 1520000 | 0.0600% | *Chroomonas* | 4966600 | 0.2000% |
| *Sphaerocystis* | 320000 | 0.0100% | *Cryptomonas* | 13750000 | 0.5700% |
| *Crucigenia* | 5320000 | 0.2200% | *Glenodinium* | 420000 | 0.0200% |
| *Pandorina* | 19255000 | 0.7900% | *Peridinium* | 653400 | 0.0300% |
| *Didymocystis* | 12890000 | 0.5300% | *Ceratium* | 280000 | 0.0100% |
| *Tetraedron* | 7425000 | 0.3100% | *Gymnodiniaceae* | 995000 | 0.0400% |
| *Tetrastrum* | 4725000 | 0.1900% | *Dinobryon* | 10246600 | 0.4200% |
| *Kitchneriella* | 840000 | 0.0300% | *Phacus* | 40000 | 0.0000% |
| *Volvox* | 1750000 | 0.0700% | *Euglena* | 2735000 | 0.1100% |
| *Dictyosphaerium* | 25720000 | 1.0600% |  |  |  |

**Table S4 FG structure**

| **Function group** | **Abundance** | **percentage** |
| --- | --- | --- |
| A | 376.42 | 0.31% |
| B | 4 | 0.00% |
| C | 10.33 | 0.01% |
| D | 690.5 | 0.57% |
| E | 537.33 | 0.44% |
| F | 3038 | 2.51% |
| G | 2616.25 | 2.16% |
| H1 | 1995.75 | 1.65% |
| J | 1538 | 1.27% |
| K | 102.67 | 0.08% |
| Kr | 247.75 | 0.20% |
| L0 | 35076.09 | 28.95% |
| LM | 14 | 0.01% |
| M | 14451.25 | 11.93% |
| MP | 11337.92 | 9.36% |
| N | 71.5 | 0.06% |
| P | 236 | 0.19% |
| Q | 3.25 | 0.00% |
| R | 6 | 0.00% |
| S2 | 36306 | 29.96% |
| SN | 3398.5 | 2.80% |
| T | 22.5 | 0.02% |
| TB | 1851.92 | 1.53% |
| TC | 767.5 | 0.63% |
| W0 | 3430.33 | 2.83% |
| W1 | 141.75 | 0.12% |
| X1 | 574.83 | 0.47% |
| X2 | 263.83 | 0.22% |
| X3 | 1226.75 | 1.01% |
| Y | 840.5 | 0.69% |

**Table S5 RDA ordering axis**

| **gen** |  |  |  |  |  | **fg** |  |  |  |  |
| --- | --- | --- | --- | --- | --- | --- | --- | --- | --- | --- |
|  | **abs(RDA1)** | |  | **abs(RDA2)** | |  | **abs(RDA1)** | |  | **abs(RDA2)** |
| **chl_a** | **0.833083** |  | **LAT** | **0.643568** |  | **chl_a** | **0.816945** |  | **LAT** | **0.539181** |
| **WIN_S_Max** | **0.472778** |  | **TN** | **0.643972** |  | **WIN_S_Max** | **0.500695** |  | **TN** | **0.496712** |
| **pH** | **0.417404** |  | **WIN_S_Max** | **0.411814** |  | **TEM** | **0.438854** |  | **TP** | **0.432129** |
| **TEM** | **0.383041** |  | **COD** | **0.408056** |  | **pH** | **0.387827** |  | **WIN_S_Max** | **0.417708** |
| **LON** | **0.337016** |  | **EC** | **0.368755** |  | **WT** | **0.338626** |  | **EC** | **0.393599** |
| **WT** | **0.314666** |  | **TDS** | **0.367576** |  | **AP** | **0.32707** |  | **TDS** | **0.392543** |
| **AP** | **0.309422** |  | **TP** | **0.341233** |  | **SD** | **0.322947** |  | **COD** | **0.381151** |
| **SD** | **0.287017** |  | **SAL** | **0.33063** |  | **LON** | **0.310926** |  | **SAL** | **0.34306** |
| **TDS** | **0.206458** |  | **SD** | **0.221561** |  | **TDS** | **0.206598** |  | **pH** | **0.260343** |
| **EC** | **0.206416** |  | **DO.** | **0.207711** |  | **EC** | **0.206471** |  | **DO.** | **0.216506** |
| **SAL** | **0.204666** |  | **AP** | **0.179392** |  | **SAL** | **0.198965** |  | **LON** | **0.1102** |
| **COD** | **0.18213** |  | **pH** | **0.145881** |  | **COD** | **0.16916** |  | **NH4_N** | **0.098984** |
| **TP** | **0.121852** |  | **WT** | **0.142803** |  | **TN** | **0.130281** |  | **AP** | **0.066385** |
| **TN** | **0.096876** |  | **RHU** | **0.115553** |  | **NH4_N** | **0.118088** |  | **chl_a** | **0.063287** |
| **LAT** | **0.057684** |  | **PRE_7D** | **0.107551** |  | **TP** | **0.09537** |  | **RHU** | **0.042313** |
| **NH4_N** | **0.055027** |  | **TEM** | **0.089869** |  | **LAT** | **0.055318** |  | **PRE_7D** | **0.041936** |
| **PRE_7D** | **0.042536** |  | **LON** | **0.086476** |  | **PRE_7D** | **0.037019** |  | **TEM** | **0.039269** |
| **RHU** | **0.039374** |  | **chl_a** | **0.048762** |  | **RHU** | **0.034125** |  | **SD** | **0.008833** |
| **DO%** | **0.002885** |  | **NH4_N** | **0.014339** |  | **DO%** | **0.02751** |  | **WT** | **0.001647** |

**Table S6 Relationships between taxa and functional groups.**

| ***fg*** | ***gen*** | ***fg*** | ***gen*** | ***fg*** | ***gen*** |
| --- | --- | --- | --- | --- | --- |
| *Lo* | *Merismopedia* | *G* | *Eudorina* | *F* | *Selenastrum* |
| *S2* | *Spirulina* | *MP* | *Coelosphaerium* | *E* | *Eunotia* |
| *M* | *Microcystis* | *J* | *Crucigenia* | *T* | *Mougeotia* |
| *MP* | *Pseudanabaena* | *F* | *Elakatothrix* | *X1* | *Ankyra* |
| *S2* | *Characium* | *X2* | *Chroomonas* | *SN* | *Cylindrospermopsis* |
| *W0* | *Oscillatoria* | *Kr* | *Melosira* | *Y* | *Glenodinium* |
| *F* | *Oocystis* | *J* | *Tetrastrum* | *F* | *Sphaerocystis* |
| *TB* | *Fragilaria* | *P* | *Coelastrum* | *M* | *Encyonema* |
| *SN* | *Anabaena* | *W1* | *Euglena* | *LM* | *Ceratium* |
| *SN* | *Raphidiopsis* | *SN* | *Nostoc* | *X2* | *Chlorogonium* |
| *Lo* | *Chroococcus* | *J* | *Lagerheimiella* | *C* | *Asterionella* |
| *G* | *Dictyosphaerium* | *M* | *Cymbella* | *TB* | *Ceratoneis* |
| *X3* | *Chlorella* | *K* | *Chaetoceros* | *K* | *Diatoma* |
| *H1* | *Aphanizomenon* | *S2* | *Dolichospermum* | *S2* | *Cymbopleura* |
| *G* | *Pandorina* | *TC* | *Gloeocapsa* | *R* | *Neidium* |
| *J* | *Desmodesmus* | *G* | *Volvox* | *X1* | *Ankistrodesmus* |
| *Y* | *Cryptomonas* | *Y* | *Cocconeis* | *P* | *Closterium* |
| *TC* | *Leptolyngbya* | *Wo* | *Chlamydomonas* | *X2* | *Gyrosigma* |
| *H1* | *Didymocystis* | *J* | *Pediastrum* | *B* | *Aulacoseira* |
| *X1* | *Monoraphidium* | *N* | *Cosmarium* | *Q* | *Frustulia* |
| *E* | *Dinobryon* | *X3* | *Schroederia* | *W1* | *Amphora* |
| *MP* | *Navicula* | *Y* | *Gymnodiniaceae* | *N* | *Staurastrum* |
| *A* | *Cyclotella* | *X3* | *Chloroccum* | *J* | *Micractinium* |
| *H1* | *Tetraedron* | *F* | *Kitchneriella* | *W1* | *Phacus* |
| *D* | *Nitzchia* | *Lo* | *Peridinium* | *J* | *Golenkinia* |
| *D* | *Ulnaria* | *SN* | *Rhabdoderma* | *J* | *Closteriopsis* |
| *S2* | *Dunaliella salina* | *TB* | *Gomphonema* | *TB* | *Stauroneis* |

**Note S1: Details for collection and analysis of biological samples.**

For qualitative analysis, use a micropipette to extract approximately 60 μL of the sample from the bottom of the sample bottle. Place the sample on a slide, cover it with a coverslip, and observe it under a microscope (×40). Each sample should be observed three times.

For quantitative analyses (**Fig.2**), the samples were thoroughly shaken before microscopic counting. Then, 0.1 ml of the mixed samples were aspirated quantitatively with a pipette and injected into the phytoplankton counting frame. The frame was covered with a coverslip and left for a few moments without bubbles before observing the samples. Finally, five counting cells were randomly selected to count the number of phytoplankton. Filamentous and spherical-like groups were estimated by cell number. The algal cell concentration (cell/L) in the water column of the sample site was calculated based on the cell count. We mainly consulted the Atlas of Freshwater Microfauna and Benthos and the Atlas of Common Aquatic Organisms in Chinese Watersheds for the taxonomic identification of algae species.

**Note S2: Taxonomy and FG composition of each lake.**

Chlorophyceae boasted the highest genera count, with 38 identified. Bacillariophyta presented 24 genera and Cyanophyceae had 16 following up. In contrast, Chrysophyceae exhibited the lowest diversity with only one genus Cysticercus, detected in specific sample sites. Cyanobacteria dominated among the phylum, constituting 82.36% (Percentage of total cells), Followed by Chlorophyceae (12.71%), Bacillariophyta (3.53%), and Dinophyta (0.1%) (**Table S2**). Notably, planktonic algae density varied significantly across the studied lakes (**Fig. 2c**), with Dongli Lake displaying the highest density at 7.46×107 cells/L and the Daihai Lake exhibited the lowest at 1.18×105 cells/L.

Noteworthy genera such as *Fragilaria* (CQH, YHY, JHH, DH, WLSH, BYD, FHEK), and *Pseudanabaena* (BYD, FKEK, DLH, GGH, HH, DH, WLSH) featured among the top 5 in seven lakes. Similarly, *Navicula* (CQH, YHY, BYD, FHEK), *Oscillatoria* (CQH, YHY, DLH, HH), and *Cryptomonas* (BYD, FHEK, HH, DH) were prominent in four lakes. Additionally, *Chlorella* (JHH, FHEK, YCYH), *Chroococcus* (CQH, YCYH, GGH), *Microcystis* (YCYH, DLH, GGH), and Spirulina (DLH, GGH, WLSH) appeared in three lakes. Thirteen other genera (*Anabaena, Aphanizomenon, Characium, Coelastrum, Desmodesmus, Dictyosphaerium, Dinobryon, Melosira, Merismopedia, Nitzchia, Oocystis, Raphidiopsis and Tetraedron*) constituted the top 5 in one or two lakes.

The MP FG featured among the top five in nine lakes (CQH, YHY, BYD, FHEK, DLH, GGH, HH, DH, WLSH), while L0 (CQH, JHH, YCYH, DLH, GGH, WLSH) and TP (CQH, YHY, JHH, BYD, FHEK, WLSH) were present in 6 lakes. S2 (YCYH, DLH, GGH, WLSH) and W0 (CQH, YHY, DLH, HH) comprised the top five in four lakes. Additionally, 14 other FGs (A, D, E, F, G, H1, J, Kr, M, P, SN, X2, X3, Y) were part of the top five in sever all lakes.

Dominance was evident among four common genera across the 11 lakes or reservoirs (**Fig. 3a**). *Cryptomonas*, *Cyclotella*, *Monoraphidium* and *Navicula* exhibited their prevalence, with each sample site hosting an exclusive number of genera ranging from 0 to 5. YCYH as the sole saline lake, boasted the highest number of exclusive genera (5 genera), including *Dunaliella salina*, *Chloroccum*, *Amphora*, *Phacus*, and *Stauroneis*. CQH exhibited the lowest total genera count among all sample sites at 15, while DLH presented the highest at 50 genera. Notably, FHEK lacked exclusive genera. Regarding FG, the 11 lakes or reservoirs collectively harbored a total of 5 FGs, namely A, MP, TB, X1 and Y. The HH featured a unique FG, while CQH had the fewest FGs at 12, and BYD and DLH possessed the highest count at 24 FGs each (**Fig. 3b**).

**Note S3: Correlation between phytoplankton and environmental factors.**

For example, a significant (P < 0.05) negative correlation was identified between *Dinobryon* and WT in the phylum Chrysophyta phylum. During the experiment, *Dinobryon* was more abundant in the early stages of sampling work (April and May), becoming rarer over time and nearly extinct in July. This pattern was shared by *Navicula*, *Didymocystis*, *Microcytosis*, and *Desmodesmus*. *Ulnaria* and *Euglena* exhibited a significant (P < 0.05) negative correlation with RHU, while *Dinobryon* and *Cryptomonoas* displayed the opposite trend, with RHU closely related to the geographical environment. Concerning FGs, M, P, G demonstrated a significant positive (P < 0.05) correlation with WT, while E showed a significant (P < 0.05) negative correlation. Additionally, E and W1 are significantly (P < 0.05) positively correlated with RHU.

Table. Metadata1 (environment factors)

| **Sample** | **LAT** | **LON** | **ELE** | **WT** | **AP** | **DO%** | **EC** | **TDS** | **SAL** | **pH** | **SD** | **NH4-N** | **TP** | **TN** | **COD** | **chl-a** | **WIN_S_Max** | **TEM** | **RHU** | **PRE_7D** |
| --- | --- | --- | --- | --- | --- | --- | --- | --- | --- | --- | --- | --- | --- | --- | --- | --- | --- | --- | --- | --- |
| **YHY1** | 40.001111 | 116.267778 | 85.9 | 16.20 | 828.30 | 120.30 | 0.30 | 0.19 | 0.14 | 8.59 | 0.45 | 0.0513 | 0.0377 | 0.0538 | 0.0423 | 0.0099 | 2.4553 | 15.7702 | 41.8723 | 0.00 |
| **YHY2** | 39.993333 | 116.275278 | 88.5 | 16.20 | 828.20 | 98.40 | 0.29 | 0.19 | 0.14 | 8.53 | 0.45 | 0.1873 | 0.1693 | 0.8407 | 0.0323 | 0.0356 | 2.4553 | 15.7702 | 41.8723 | 0.00 |
| **CQH1** | 39.794722 | 116.080833 | 128.9 | 14.50 | 825.10 | 121.30 | 0.07 | 0.03 | 0.02 | 8.29 | 0.50 | 0.0920 | 0.0120 | 2.6977 | 0.9763 | 0.0072 | 2.0511 | 15.8426 | 38.9362 | 0.00 |
| **CQH2** | 39.798056 | 116.079444 | 126.5 | 13.80 | 825.10 | 128.40 | 0.67 | 0.43 | 0.33 | 8.24 | 0.45 | 0.0565 | 0.0153 | 2.5280 | 0.8625 | 0.0076 | 2.0511 | 15.8426 | 38.9362 | 0.00 |
| **JHH1** | 40.181944 | 117.306389 | 115.7 | 10.20 | 826.10 | 133.90 | 0.40 | 0.26 | 0.19 | 8.55 | 2.30 | 0.0518 | 0.0103 | 1.6127 | 6.4637 | 0.0081 | 2.7340 | 14.5766 | 39.7660 | 0.00 |
| **JHH2** | 40.177222 | 117.299722 | 114.8 | 9.80 | 825.80 | 125.90 | 0.40 | 0.26 | 0.19 | 8.73 | 2.30 | 0.0778 | 0.0110 | 1.2517 | 6.6320 | 0.0080 | 2.7340 | 14.5766 | 39.7660 | 0.00 |
| **JHH3** | 40.180278 | 117.298056 | 117.7 | 10.30 | 825.80 | 123.70 | 0.40 | 0.11 | 0.08 | 8.54 | 2.30 | 0.0897 | 0.0117 | 1.1370 | 6.6913 | 0.0072 | 2.7340 | 14.5766 | 39.7660 | 0.00 |
| **BYD1** | 38.935833 | 116.014722 | 7.4 | 17.60 | 823.90 | 140.00 | 0.80 | 0.52 | 0.39 | 8.59 | 1.20 | 0.0833 | 0.0060 | 0.9810 | 1.7753 | 0.0094 | 2.6370 | 13.9630 | 61.8913 | 35.00 |
| **BYD2** | 38.947222 | 115.996111 | 9.0 | 17.10 | 824.00 | 138.70 | 0.74 | 0.48 | 0.37 | 8.67 | 1.20 | 0.0947 | 0.0030 | 1.6697 | 2.1217 | 0.0067 | 2.6370 | 13.9630 | 61.8913 | 35.00 |
| **BYD3** | 38.950000 | 115.986389 | 8.6 | 18.00 | 823.50 | 121.50 | 0.78 | 0.51 | 0.38 | 8.34 | 1.10 | 0.0970 | 0.0227 | 0.8057 | 4.2403 | 0.0074 | 2.6370 | 13.9630 | 61.8913 | 35.00 |
| **BYD4** | 38.923611 | 115.980278 | 4.7 | 17.40 | 822.80 | 101.30 | 0.84 | 0.54 | 0.41 | 8.17 | 1.20 | 0.0957 | 0.0050 | 0.4493 | 1.8723 | 0.0098 | 2.6370 | 13.9630 | 61.8913 | 35.00 |
| **BYD5** | 38.880278 | 116.049167 | 11.8 | 17.50 | 814.20 | 139.30 | 0.93 | 0.60 | 0.46 | 8.64 | 1.70 | 0.0290 | 0.0140 | 0.3243 | 4.1277 | 0.0039 | 3.3978 | 14.0630 | 59.1087 | 35.40 |
| **BYD6** | 38.898333 | 116.058056 | 2.8 | 17.80 | 814.00 | 115.20 | 0.94 | 0.61 | 0.47 | 8.39 | 1.70 | 0.0957 | 0.1600 | 0.4750 | 4.0880 | 0.0052 | 3.3978 | 14.0630 | 59.1087 | 35.40 |
| **BYD7** | 38.980556 | 116.014722 | 10.1 | 17.50 | 815.10 | 133.80 | 0.62 | 0.45 | 0.30 | 8.94 | 1.00 | 1.3333 | 0.2233 | 4.1657 | 2.6693 | 0.0066 | 3.0978 | 13.4913 | 62.5000 | 18.10 |
| **FHEK** | 37.981667 | 112.376944 | 859.3 | 10.00 | 692.60 | 73.20 | 0.72 | 0.47 | 0.36 | 8.41 | 1.70 | 0.3640 | 0.0120 | 2.5700 | 2.6700 | 0.0006 | 2.2424 | 13.5478 | 73.5761 | 46.10 |
| **YCYH1** | 35.000556 | 111.037222 | 289.0 | 26.00 | 798.00 | 34.80 | 95.70 | 62.10 | 68.83 | 8.33 | 0.50 | 0.1170 | 1.1800 | 13.7000 | 200.0000 | 0.0120 | 2.3239 | 26.2978 | 47.6739 | 0.70 |
| **YCYH2** | 34.985556 | 111.020278 | 322.8 | 29.30 | 810.10 | 189.60 | 94.10 | 61.10 | 67.41 | 8.31 | 0.50 | 0.2320 | 1.1700 | 7.3300 | 181.0000 | 0.0360 | 2.3239 | 26.2978 | 47.6739 | 0.70 |
| **YCYH3** | 34.966944 | 110.851389 | 332.7 | 27.60 | 806.70 | 113.20 | 40.05 | 25.86 | 25.31 | 8.75 | 0.23 | 0.5280 | 0.4700 | 19.8000 | 51.9000 | 0.0120 | 2.3239 | 26.2978 | 47.6739 | 0.70 |
| **YCYH4** | 34.962778 | 110.943333 | 321.7 | 27.60 | 805.60 | 104.30 | 39.12 | 25.41 | 24.84 | 8.74 | 0.19 | 0.4260 | 0.9500 | 4.3500 | 163.0000 | 0.0530 | 2.3239 | 26.2978 | 47.6739 | 0.70 |
| **YCYH5** | 34.936111 | 110.897778 | 320.4 | 34.00 | 809.20 | 216.50 | 37.61 | 24.43 | 23.61 | 8.75 | 0.26 | 0.4260 | 0.3700 | 16.8000 | 50.3000 | 0.0100 | 2.3239 | 26.2978 | 47.6739 | 0.70 |
| **YCYH6** | 34.932222 | 110.861389 | 346.0 | 30.20 | 830.30 | 59.10 | 4.27 | 2.78 | 2.25 | 8.52 | 0.45 | 4.7400 | 1.3700 | 18.2000 | 14.2000 | 0.0110 | 2.3239 | 26.2978 | 47.6739 | 0.70 |
| **YCYH7** | 35.013333 | 111.056944 | 299.3 | 26.30 | 790.50 | 71.40 | 95.00 | 61.70 | 68.32 | 8.37 | 0.27 | 0.0860 | 0.6500 | 15.1000 | 88.5000 | 0.0300 | 2.3239 | 26.2978 | 47.6739 | 0.70 |
| **DLH1** | 39.167069 | 117.477190 | -2.8 | 30.10 | 836.10 | 128.30 | 4.11 | 2.67 | 2.16 | 9.27 | 0.37 | 0.4377 | 0.2667 | 7.1340 | -0.0133 | 0.0733 | 3.5458 | 29.5125 | 58.9583 | 14.00 |
| **DLH2** | 39.167008 | 117.471612 | -0.2 | 30.30 | 833.50 | 112.50 | 4.70 | 3.05 | 2.49 | 9.05 | 0.26 | 1.1737 | 0.7793 | 3.6957 | -0.2690 | 0.1179 | 3.5458 | 29.5125 | 58.9583 | 14.00 |
| **GGH1** | 38.921596 | 117.515508 | -6.7 | 31.70 | 836.80 | 39.70 | 8.23 | 5.36 | 4.53 | 8.64 | 0.45 | 1.5847 | 0.1690 | 8.7967 | 0.0190 | 0.0493 | 3.5458 | 29.5125 | 58.9583 | 14.00 |
| **GGH2** | 38.920431 | 117.517883 | -5.7 | 32.20 | 834.90 | 12.80 | 8.20 | 5.34 | 4.51 | 8.50 | 0.50 | 1.8533 | 0.2247 | 10.3910 | 0.0183 | 0.0449 | 3.5458 | 29.5125 | 58.9583 | 14.00 |
| **HH** | 39.153429 | 117.181087 | 0.6 | 31.80 | 834.70 | 90.80 | 0.59 | 0.38 | 6.28 | 8.37 | 0.30 | 0.3520 | 0.0680 | 3.9097 | 0.0607 | 0.0439 | 3.5458 | 29.5125 | 58.9583 | 14.00 |
| **DH1** | 40.592225 | 112.646153 | 1211.9 | 28.50 | 731.70 | 110.60 | 27.46 | 17.90 | 16.85 | 8.71 | 0.20 | 3.4447 | 0.1067 | 0.8730 | 4.1867 | 0.0043 | 2.8709 | 23.0455 | 77.7636 | 17.80 |
| **DH2** | 40.585002 | 112.655052 | 1212.8 | 26.80 | 736.10 | 103.20 | 28.24 | 18.38 | 17.36 | 8.83 | 2.00 | 3.2837 | 0.0660 | 0.2817 | 4.3037 | 0.0011 | 2.8709 | 23.0455 | 77.7636 | 17.80 |
| **DH3** | 40.605935 | 112.731652 | 1216.9 | 26.60 | 734.20 | 95.90 | 28.22 | 18.35 | 17.35 | 8.85 | 2.10 | 3.3963 | 0.0060 | 0.3647 | 4.4027 | 0.0007 | 2.8709 | 23.0455 | 77.7636 | 17.80 |
| **DH4** | 40.589824 | 112.666927 | 1214.1 | 26.70 | 734.00 | 92.90 | 28.22 | 18.35 | 17.35 | 8.86 | 2.10 | 3.5210 | 0.0737 | 2.1283 | 4.3973 | 0.0031 | 2.8709 | 23.0455 | 77.7636 | 17.80 |
| **DH5** | 40.592793 | 112.662737 | 1217.9 | 26.90 | 732.90 | 90.30 | 28.22 | 18.35 | 17.35 | 8.86 | 2.10 | 3.2267 | 0.0583 | 1.3767 | 4.3860 | 0.0015 | 2.8709 | 23.0455 | 77.7636 | 17.80 |
| **DH6** | 40.593021 | 112.655314 | 1215.1 | 28.00 | 733.30 | 81.20 | 27.94 | 18.20 | 17.17 | 8.82 | 0.15 | 3.9253 | 0.1083 | 1.7773 | 4.1790 | 0.0043 | 2.8709 | 23.0455 | 77.7636 | 17.80 |
| **WLSH1** | 40.825695 | 108.763263 | 1017.8 | 27.70 | 736.00 | 65.60 | 4.22 | 2.73 | 2.23 | 8.98 | 0.80 | 0.3943 | 0.0350 | 0.1287 | 3.2433 | 0.0108 | 3.4945 | 28.2727 | 52.6545 | 0.30 |
| **WLSH2** | 40.829468 | 108.767932 | 1023.8 | 28.20 | 740.30 | 71.80 | 4.38 | 2.84 | 2.32 | 9.03 | 0.90 | 0.3250 | 0.0380 | 0.5727 | 3.2277 | 0.0114 | 3.4945 | 28.2727 | 52.6545 | 0.30 |
| **WLSH3** | 40.831028 | 108.767943 | 1024.9 | 27.10 | 738.40 | 72.10 | 4.35 | 2.82 | 2.30 | 9.35 | 1.20 | 0.3977 | 0.0320 | 0.5660 | 3.2683 | 0.0093 | 3.4945 | 28.2727 | 52.6545 | 0.30 |
| **WLSH4** | 40.847142 | 108.750408 | 1020.9 | 28.40 | 748.90 | 80.00 | 2.71 | 1.76 | 1.40 | 8.27 | 0.80 | 0.3920 | 0.0303 | 0.0203 | 3.2470 | 0.0136 | 3.4945 | 28.2727 | 52.6545 | 0.30 |
| **WLSH5** | 40.859064 | 108.748735 | 1038.4 | 29.10 | 747.30 | 91.20 | 3.23 | 2.17 | 1.73 | 8.56 | 0.80 | 0.3837 | 0.0310 | 0.2873 | 3.2260 | 0.0137 | 3.4945 | 28.2727 | 52.6545 | 0.30 |
| **WLSH6** | 40.828373 | 108.762462 | 1022.5 | 27.20 | 746.00 | 53.80 | 3.79 | 2.49 | 1.99 | 8.73 | 0.60 | 0.3590 | 0.0290 | 0.1843 | 3.2567 | 0.0109 | 3.4945 | 28.2727 | 52.6545 | 0.30 |

Table. Metadata2 (Phytoplankton community /20000 cell/L)

| *fg* | *gen* | CQH1 | CQH2 | YHY1 | YHY2 | JHH1 | JHH2 | JHH3 | BYD1 | BYD2 | BYD3 | BYD4 | BYD5 | BYD6 | BYD7 | FHEK | YCYH1 | YCYH2 | YCYH3 | YCYH4 | YCYH5 | YCYH6 | YCYH7 | DLH1 | DLH2 | GGH1 | GGH2 | HH | DH1 | DH2 | DH3 | DH4 | DH5 | DH6 | WLSH1 | WLSH2 | WLSH3 | WLSH4 | WLSH5 | WLSH6 |
| --- | --- | --- | --- | --- | --- | --- | --- | --- | --- | --- | --- | --- | --- | --- | --- | --- | --- | --- | --- | --- | --- | --- | --- | --- | --- | --- | --- | --- | --- | --- | --- | --- | --- | --- | --- | --- | --- | --- | --- | --- |
| *Lo* | *Merismopedia* | 0 | 0 | 8 | 0 | 8 | 14 | 16 | 0 | 0 | 4 | 4 | 0 | 4 | 0 | 0 | 0 | 0 | 0 | 0 | 0 | 0 | 0 | 3920 | 24200 | 210 | 20 | 0 | 0 | 0 | 0 | 0 | 0 | 0 | 676 | 224 | 48 | 1636 | 2430 | 134 |
| *S2* | *Spirulina* | 0 | 0 | 0 | 80 | 0 | 0 | 0 | 0 | 0 | 0 | 0 | 0 | 0 | 0 | 0 | 0 | 0 | 0 | 0 | 0 | 0 | 0 | 1300 | 27875 | 210 | 30 | 0 | 0 | 0 | 0 | 0 | 0 | 0 | 40 | 0 | 0 | 190 | 1480 | 0 |
| *M* | *Microcystis* | 0 | 0 | 70 | 7 | 0 | 0 | 60 | 0 | 0 | 0 | 0 | 0 | 0 | 0 | 0 | 0 | 0 | 0 | 0 | 0 | 780 | 0 | 2750 | 1862.5 | 2292 | 6420 | 40 | 0 | 0 | 0 | 0 | 0 | 0 | 0 | 30 | 0 | 0 | 30 | 0 |
| *MP* | *Pseudanabaena* | 0 | 0 | 0 | 0 | 0 | 0 | 0 | 0 | 0 | 3 | 120 | 25 | 40 | 0 | 6.67 | 0 | 0 | 40 | 86 | 35 | 10 | 0 | 5525 | 900 | 1270 | 460 | 561 | 60 | 0 | 0 | 0 | 0 | 0 | 488 | 310 | 80 | 104 | 371 | 59 |
| *S2* | *Characium* | 0 | 0 | 0 | 0 | 0 | 0 | 0 | 0 | 0 | 0 | 0 | 0 | 0 | 0 | 0 | 1057 | 3248 | 0 | 0 | 0 | 0 | 400 | 0 | 0 | 0 | 0 | 0 | 0 | 0 | 0 | 0 | 0 | 0 | 4 | 4 | 7 | 1 | 0 | 0 |
| *W0* | *Oscillatoria* | 0 | 23 | 72 | 320 | 0 | 23 | 34 | 0 | 0 | 0 | 0 | 0 | 20 | 0 | 0 | 18 | 0 | 0 | 0 | 0 | 0 | 0 | 2350 | 100 | 0 | 50 | 210 | 0 | 0 | 0 | 0 | 0 | 0 | 0 | 20 | 0 | 40 | 70 | 0 |
| *F* | *Oocystis* | 0 | 0 | 0 | 0 | 0 | 0 | 4 | 0 | 0 | 0 | 0 | 0 | 0 | 0 | 0 | 2 | 0 | 821 | 963 | 612 | 11 | 2 | 165 | 20 | 47 | 17 | 1 | 0 | 0 | 0 | 0 | 0 | 0 | 0 | 4 | 0 | 4 | 18 | 5 |
| *TB* | *Fragilaria* | 5 | 14 | 113 | 106 | 104 | 115 | 129 | 12 | 10 | 36 | 5 | 9 | 5 | 50 | 25.67 | 0 | 0 | 0 | 0 | 0 | 0 | 0 | 25 | 70 | 5 | 1 | 41 | 0 | 2 | 0 | 0 | 0 | 2 | 33 | 6 | 4 | 206 | 644 | 39 |
| *SN* | *Anabaena* | 0 | 0 | 530 | 890 | 17 | 0 | 0 | 0 | 0 | 0 | 0 | 0 | 0 | 0 | 0 | 0 | 0 | 0 | 0 | 0 | 0 | 0 | 100 | 0 | 0 | 0 | 43 | 0 | 0 | 0 | 0 | 0 | 0 | 0 | 15 | 30 | 7 | 0 | 23 |
| *SN* | *Raphidiopsis* | 0 | 0 | 0 | 0 | 0 | 0 | 0 | 0 | 0 | 0 | 0 | 0 | 0 | 0 | 0 | 0 | 0 | 0 | 0 | 0 | 0 | 0 | 0 | 0 | 0 | 0 | 1590 | 0 | 0 | 0 | 0 | 0 | 0 | 0 | 0 | 0 | 0 | 0 | 0 |
| *Lo* | *Chroococcus* | 15 | 5 | 5 | 4 | 7 | 19 | 10 | 1 | 7 | 13 | 0 | 8 | 8 | 0 | 0.67 | 0 | 0 | 0 | 0 | 0 | 724 | 0 | 10 | 48.75 | 170 | 78 | 22 | 0 | 0 | 0 | 0 | 0 | 0 | 80 | 48 | 6 | 45 | 125 | 28 |
| *G* | *Dictyosphaerium* | 0 | 0 | 0 | 0 | 0 | 0 | 0 | 0 | 0 | 0 | 0 | 0 | 0 | 0 | 0 | 0 | 0 | 0 | 0 | 0 | 0 | 0 | 0 | 10 | 48 | 0 | 0 | 0 | 0 | 0 | 0 | 0 | 0 | 408 | 144 | 0 | 168 | 320 | 188 |
| *X3* | *Chlorella* | 4 | 5 | 11 | 10 | 25 | 16 | 21 | 6 | 1 | 4 | 5 | 6 | 7 | 5 | 5 | 89 | 72 | 424 | 62 | 0 | 0 | 23 | 95 | 113.75 | 77 | 24 | 14 | 0 | 0 | 0 | 0 | 0 | 0 | 0 | 0 | 0 | 0 | 0 | 0 |
| *H1* | *Aphanizomenon* | 0 | 0 | 20 | 75 | 265 | 300 | 260 | 0 | 15 | 0 | 20 | 25 | 0 | 0 | 0 | 0 | 0 | 0 | 0 | 0 | 0 | 0 | 0 | 0 | 0 | 0 | 0 | 0 | 0 | 0 | 0 | 0 | 0 | 0 | 0 | 0 | 0 | 0 | 0 |
| *G* | *Pandorina* | 0 | 0 | 0 | 0 | 0 | 0 | 0 | 0 | 0 | 0 | 0 | 0 | 0 | 0 | 0 | 0 | 0 | 7 | 0 | 0 | 80 | 0 | 0 | 23.75 | 488 | 184 | 0 | 0 | 0 | 0 | 0 | 0 | 0 | 0 | 48 | 0 | 0 | 132 | 0 |
| *J* | *Desmodesmus* | 4 | 8 | 2 | 18 | 0 | 0 | 0 | 14 | 24 | 16 | 12 | 17 | 6 | 12 | 0 | 0 | 0 | 0 | 0 | 0 | 4 | 0 | 200 | 173.75 | 50 | 20 | 72 | 0 | 0 | 0 | 0 | 2 | 0 | 12 | 2 | 6 | 100 | 78 | 6 |
| *Y* | *Cryptomonas* | 2 | 3 | 0 | 3 | 4 | 5 | 4 | 12 | 21 | 36 | 30 | 6 | 22 | 31 | 9 | 5 | 0 | 29 | 10 | 9 | 1 | 2 | 150 | 27.5 | 80 | 27 | 70 | 1 | 1 | 1 | 1 | 0 | 1 | 10 | 21 | 11 | 12 | 18 | 12 |
| *TC* | *Leptolyngbya* | 0 | 0 | 0 | 0 | 0 | 0 | 0 | 0 | 0 | 0 | 0 | 0 | 0 | 20 | 0 | 0 | 0 | 0 | 0 | 0 | 0 | 0 | 325 | 212.5 | 50 | 50 | 20 | 0 | 0 | 0 | 0 | 0 | 0 | 0 | 0 | 0 | 0 | 0 | 0 |
| *H1* | *Didymocystis* | 0 | 0 | 0 | 0 | 0 | 0 | 0 | 0 | 0 | 0 | 0 | 0 | 0 | 0 | 0 | 0 | 0 | 164 | 104 | 126 | 0 | 0 | 160 | 22.5 | 2 | 0 | 0 | 0 | 0 | 0 | 0 | 0 | 0 | 12 | 10 | 6 | 20 | 6 | 12 |
| *X1* | *Monoraphidium* | 2 | 5 | 3 | 7 | 8 | 5 | 2 | 6 | 8 | 5 | 5 | 14 | 17 | 2 | 0.33 | 0 | 0 | 0 | 0 | 0 | 2 | 0 | 100 | 56.25 | 7 | 4 | 5 | 0 | 1 | 0 | 0 | 0 | 0 | 59 | 76 | 45 | 23 | 31 | 50 |
| *E* | *Dinobryon* | 0 | 0 | 4 | 7 | 22 | 27 | 17 | 147 | 82 | 40 | 98 | 31 | 35 | 2 | 0.33 | 0 | 0 | 0 | 0 | 0 | 0 | 0 | 0 | 0 | 0 | 0 | 0 | 0 | 0 | 0 | 0 | 0 | 0 | 0 | 0 | 0 | 0 | 0 | 0 |
| *MP* | *Navicula* | 11 | 15 | 43 | 163 | 12 | 28 | 15 | 8 | 14 | 32 | 4 | 10 | 9 | 35 | 4 | 0 | 0 | 3 | 7 | 0 | 16 | 0 | 0 | 26.25 | 7 | 3 | 5 | 0 | 1 | 0 | 1 | 1 | 1 | 5 | 4 | 1 | 6 | 12 | 6 |
| *A* | *Cyclotella* | 26 | 11 | 13 | 21 | 16 | 6 | 17 | 3 | 4 | 7 | 6 | 9 | 10 | 7 | 0.67 | 0 | 0 | 2 | 7 | 1 | 0 | 0 | 40 | 23.75 | 29 | 15 | 29 | 0 | 1 | 0 | 0 | 0 | 0 | 8 | 3 | 1 | 32 | 16 | 12 |
| *H1* | *Tetraedron* | 0 | 0 | 2 | 0 | 22 | 23 | 41 | 8 | 4 | 3 | 0 | 6 | 5 | 1 | 0 | 2 | 0 | 0 | 0 | 0 | 0 | 8 | 125 | 56.25 | 8 | 10 | 2 | 0 | 0 | 0 | 0 | 0 | 0 | 4 | 6 | 4 | 10 | 13 | 8 |
| *D* | *Nitzchia* | 0 | 0 | 0 | 0 | 0 | 0 | 0 | 0 | 0 | 0 | 9 | 5 | 4 | 1 | 0 | 0 | 2 | 38 | 13 | 7 | 0 | 0 | 190 | 41.25 | 1 | 0 | 3 | 0 | 0 | 0 | 0 | 1 | 12 | 0 | 0 | 0 | 2 | 20 | 7 |
| *D* | *Ulnaria* | 0 | 0 | 0 | 0 | 0 | 0 | 0 | 0 | 0 | 0 | 0 | 0 | 0 | 0 | 0 | 0 | 0 | 0 | 0 | 0 | 0 | 0 | 10 | 1.25 | 0 | 0 | 8 | 0 | 0 | 0 | 1 | 0 | 0 | 31 | 9 | 6 | 156 | 100 | 12 |
| *S2* | *Dunaliella salina* | 0 | 0 | 0 | 0 | 0 | 0 | 0 | 0 | 0 | 0 | 0 | 0 | 0 | 0 | 0 | 0 | 0 | 65 | 63 | 155 | 0 | 0 | 0 | 0 | 0 | 0 | 0 | 0 | 0 | 0 | 0 | 0 | 0 | 0 | 0 | 0 | 0 | 0 | 0 |
| *G* | *Eudorina* | 0 | 0 | 0 | 0 | 0 | 0 | 0 | 16 | 0 | 0 | 0 | 0 | 0 | 0 | 0 | 0 | 0 | 8 | 0 | 0 | 0 | 0 | 0 | 0 | 0 | 0 | 0 | 0 | 0 | 0 | 0 | 0 | 0 | 0 | 64 | 0 | 128 | 64 | 0 |
| *MP* | *Coelosphaerium* | 0 | 0 | 0 | 5 | 0 | 0 | 0 | 0 | 0 | 0 | 0 | 0 | 0 | 0 | 0 | 0 | 0 | 0 | 0 | 0 | 0 | 0 | 0 | 0 | 0 | 0 | 0 | 0 | 0 | 0 | 0 | 0 | 0 | 0 | 0 | 16 | 34 | 211 | 10 |
| *J* | *Crucigenia* | 0 | 0 | 0 | 0 | 0 | 0 | 0 | 0 | 0 | 5 | 2 | 30 | 4 | 0 | 0 | 0 | 0 | 5 | 8 | 0 | 0 | 0 | 60 | 140 | 0 | 0 | 4 | 0 | 0 | 0 | 0 | 0 | 0 | 0 | 0 | 0 | 4 | 0 | 4 |
| *F* | *Elakatothrix* | 0 | 0 | 0 | 0 | 0 | 0 | 0 | 0 | 0 | 0 | 0 | 0 | 0 | 0 | 0 | 0 | 0 | 0 | 0 | 0 | 0 | 0 | 50 | 10 | 0 | 0 | 0 | 11 | 12 | 13 | 47 | 58 | 55 | 3 | 0 | 0 | 0 | 0 | 0 |
| *X2* | *Chroomonas* | 0 | 0 | 0 | 0 | 0 | 0 | 0 | 20 | 11 | 18 | 5 | 10 | 14 | 6 | 6.33 | 0 | 0 | 0 | 0 | 0 | 0 | 0 | 0 | 0 | 0 | 0 | 0 | 0 | 0 | 3 | 4 | 5 | 13 | 20 | 43 | 40 | 7 | 11 | 12 |
| *Kr* | *Melosira* | 1 | 1 | 35 | 96 | 0 | 0 | 0 | 0 | 0 | 20 | 9 | 0 | 0 | 4 | 2 | 0 | 0 | 0 | 0 | 0 | 0 | 0 | 50 | 3.75 | 0 | 0 | 26 | 0 | 0 | 0 | 0 | 0 | 0 | 0 | 0 | 0 | 0 | 0 | 0 |
| *J* | *Tetrastrum* | 0 | 0 | 0 | 0 | 0 | 0 | 0 | 0 | 0 | 0 | 15 | 0 | 0 | 0 | 0 | 0 | 0 | 0 | 0 | 0 | 0 | 0 | 200 | 21.25 | 0 | 0 | 0 | 0 | 0 | 0 | 0 | 0 | 0 | 0 | 0 | 0 | 0 | 0 | 0 |
| *P* | *Coelastrum* | 0 | 0 | 0 | 0 | 0 | 0 | 0 | 0 | 0 | 0 | 0 | 0 | 0 | 0 | 0 | 0 | 0 | 0 | 0 | 0 | 0 | 0 | 80 | 15 | 40 | 24 | 40 | 0 | 0 | 8 | 8 | 0 | 0 | 0 | 0 | 0 | 16 | 0 | 0 |
| *W1* | *Euglena* | 1 | 2 | 0 | 0 | 2 | 3 | 0 | 5 | 8 | 4 | 5 | 1 | 3 | 4 | 1 | 1 | 1 | 0 | 0 | 0 | 4 | 3 | 35 | 33.75 | 6 | 2 | 3 | 0 | 0 | 0 | 0 | 0 | 0 | 1 | 1 | 0 | 1 | 6 | 0 |
| *SN* | *Nostoc* | 0 | 0 | 0 | 5 | 0 | 0 | 0 | 0 | 0 | 0 | 0 | 0 | 0 | 0 | 0 | 0 | 0 | 0 | 0 | 0 | 0 | 0 | 40 | 46.25 | 11 | 0 | 0 | 0 | 0 | 0 | 0 | 0 | 0 | 0 | 0 | 0 | 0 | 0 | 0 |
| *J* | *Lagerheimiella* | 0 | 0 | 0 | 0 | 0 | 0 | 0 | 0 | 1 | 1 | 0 | 1 | 0 | 1 | 0 | 0 | 0 | 0 | 0 | 0 | 0 | 0 | 40 | 10 | 1 | 1 | 1 | 0 | 0 | 0 | 0 | 0 | 0 | 7 | 6 | 2 | 6 | 16 | 3 |
| *M* | *Cymbella* | 0 | 2 | 9 | 13 | 0 | 1 | 0 | 0 | 4 | 8 | 4 | 3 | 4 | 1 | 0 | 0 | 0 | 2 | 2 | 2 | 3 | 0 | 5 | 3.75 | 3 | 0 | 3 | 1 | 0 | 1 | 0 | 1 | 2 | 0 | 0 | 0 | 9 | 4 | 5 |
| *K* | *Chaetoceros* | 0 | 0 | 0 | 0 | 0 | 0 | 0 | 0 | 0 | 0 | 0 | 0 | 0 | 0 | 0 | 0 | 0 | 0 | 0 | 0 | 0 | 0 | 0 | 0 | 54 | 40 | 0 | 0 | 0 | 0 | 0 | 0 | 0 | 0 | 0 | 0 | 0 | 0 | 0 |
| *S2* | *Dolichospermum* | 0 | 0 | 0 | 0 | 0 | 0 | 0 | 0 | 0 | 0 | 0 | 0 | 0 | 0 | 0 | 0 | 0 | 0 | 0 | 0 | 0 | 0 | 0 | 0 | 0 | 0 | 0 | 0 | 0 | 0 | 0 | 0 | 0 | 0 | 0 | 0 | 0 | 90 | 0 |
| *TC* | *Gloeocapsa* | 0 | 0 | 0 | 0 | 0 | 0 | 0 | 0 | 0 | 0 | 0 | 0 | 16 | 0 | 0 | 30 | 0 | 14 | 0 | 0 | 0 | 30 | 0 | 0 | 0 | 0 | 0 | 0 | 0 | 0 | 0 | 0 | 0 | 0 | 0 | 0 | 0 | 0 | 0 |
| *G* | *Volvox* | 0 | 0 | 0 | 0 | 0 | 0 | 0 | 0 | 0 | 0 | 0 | 0 | 0 | 0 | 0 | 0 | 0 | 0 | 0 | 0 | 0 | 0 | 0 | 87.5 | 0 | 0 | 0 | 0 | 0 | 0 | 0 | 0 | 0 | 0 | 0 | 0 | 0 | 0 | 0 |
| *Y* | *Cocconeis* | 1 | 1 | 1 | 0 | 0 | 1 | 0 | 0 | 0 | 1 | 0 | 0 | 0 | 0 | 0 | 0 | 0 | 0 | 0 | 0 | 0 | 0 | 50 | 6.25 | 7 | 1 | 0 | 0 | 0 | 0 | 0 | 0 | 0 | 0 | 0 | 0 | 2 | 6 | 5 |
| *Wo* | *Chlamydomonas* | 0 | 0 | 0 | 0 | 0 | 0 | 0 | 2 | 2 | 5 | 6 | 0 | 0 | 1 | 0.33 | 0 | 0 | 0 | 0 | 0 | 4 | 0 | 20 | 20 | 3 | 3 | 4 | 0 | 0 | 0 | 0 | 0 | 0 | 0 | 0 | 1 | 1 | 4 | 4 |
| *J* | *Pediastrum* | 0 | 0 | 0 | 8 | 0 | 0 | 0 | 0 | 8 | 0 | 0 | 0 | 0 | 0 | 0 | 0 | 0 | 0 | 0 | 0 | 0 | 0 | 0 | 60 | 0 | 0 | 0 | 0 | 0 | 0 | 0 | 0 | 0 | 0 | 0 | 0 | 0 | 0 | 0 |
| *N* | *Cosmarium* | 0 | 0 | 0 | 0 | 0 | 0 | 0 | 0 | 0 | 0 | 0 | 0 | 0 | 0 | 0 | 0 | 0 | 52 | 0 | 0 | 0 | 0 | 10 | 2.5 | 1 | 0 | 0 | 0 | 0 | 0 | 0 | 0 | 0 | 0 | 0 | 2 | 0 | 0 | 2 |
| *X3* | *Schroederia* | 0 | 0 | 0 | 0 | 0 | 0 | 0 | 0 | 0 | 0 | 0 | 0 | 0 | 0 | 0 | 0 | 0 | 4 | 23 | 16 | 0 | 0 | 5 | 0 | 0 | 0 | 0 | 1 | 1 | 1 | 3 | 0 | 0 | 0 | 0 | 0 | 0 | 0 | 0 |
| *Y* | *Gymnodiniaceae* | 0 | 0 | 0 | 0 | 0 | 0 | 1 | 0 | 0 | 0 | 2 | 1 | 6 | 1 | 0 | 0 | 0 | 0 | 0 | 0 | 0 | 0 | 10 | 3.75 | 0 | 0 | 0 | 0 | 0 | 0 | 0 | 0 | 0 | 2 | 6 | 1 | 4 | 11 | 1 |
| *X3* | *Chloroccum* | 0 | 0 | 0 | 0 | 0 | 0 | 0 | 0 | 0 | 0 | 0 | 0 | 0 | 0 | 0 | 11 | 0 | 0 | 0 | 0 | 0 | 37 | 0 | 0 | 0 | 0 | 0 | 0 | 0 | 0 | 0 | 0 | 0 | 0 | 0 | 0 | 0 | 0 | 0 |
| *F* | *Kitchneriella* | 0 | 0 | 0 | 0 | 0 | 0 | 0 | 0 | 3 | 3 | 0 | 0 | 2 | 0 | 1 | 0 | 0 | 0 | 0 | 0 | 0 | 0 | 0 | 20 | 5 | 8 | 0 | 0 | 0 | 0 | 0 | 0 | 0 | 0 | 0 | 0 | 0 | 0 | 0 |
| *Lo* | *Peridinium* | 11 | 1 | 1 | 0 | 0 | 0 | 0 | 7 | 4 | 6 | 1 | 0 | 0 | 0 | 1.67 | 0 | 0 | 0 | 0 | 0 | 0 | 0 | 0 | 0 | 0 | 0 | 0 | 0 | 0 | 0 | 0 | 0 | 0 | 0 | 0 | 0 | 0 | 0 | 0 |
| *SN* | *Rhabdoderma* | 0 | 0 | 0 | 0 | 0 | 0 | 0 | 0 | 0 | 0 | 0 | 5 | 5 | 1 | 0 | 0 | 0 | 0 | 0 | 0 | 0 | 0 | 0 | 6.25 | 8 | 2 | 2 | 1 | 0 | 0 | 0 | 0 | 0 | 0 | 0 | 0 | 0 | 0 | 0 |
| *TB* | *Gomphonema* | 0 | 1 | 0 | 0 | 0 | 0 | 0 | 0 | 0 | 0 | 2 | 1 | 1 | 5 | 0 | 0 | 0 | 9 | 0 | 0 | 0 | 0 | 5 | 1.25 | 0 | 0 | 0 | 0 | 0 | 0 | 0 | 0 | 0 | 0 | 0 | 0 | 0 | 0 | 0 |
| *F* | *Selenastrum* | 0 | 0 | 0 | 0 | 0 | 0 | 0 | 0 | 0 | 0 | 0 | 0 | 0 | 0 | 0 | 0 | 0 | 0 | 0 | 0 | 0 | 0 | 25 | 0 | 0 | 0 | 0 | 0 | 0 | 0 | 0 | 0 | 0 | 0 | 0 | 0 | 0 | 0 | 0 |
| *E* | *Eunotia* | 0 | 0 | 0 | 0 | 0 | 0 | 0 | 0 | 0 | 0 | 0 | 0 | 0 | 0 | 0 | 0 | 0 | 0 | 0 | 0 | 0 | 0 | 25 | 0 | 0 | 0 | 0 | 0 | 0 | 0 | 0 | 0 | 0 | 0 | 0 | 0 | 0 | 0 | 0 |
| *T* | *Mougeotia* | 0 | 0 | 0 | 0 | 0 | 0 | 0 | 0 | 0 | 0 | 0 | 0 | 0 | 0 | 0 | 0 | 0 | 0 | 0 | 0 | 0 | 0 | 0 | 2.5 | 0 | 0 | 0 | 12 | 0 | 0 | 0 | 0 | 0 | 0 | 0 | 0 | 0 | 0 | 8 |
| *X1* | *Ankyra* | 0 | 0 | 0 | 0 | 0 | 0 | 0 | 0 | 0 | 0 | 0 | 0 | 0 | 0 | 0 | 0 | 0 | 0 | 0 | 0 | 0 | 0 | 20 | 1.25 | 0 | 0 | 0 | 0 | 0 | 0 | 0 | 0 | 0 | 0 | 0 | 0 | 0 | 0 | 0 |
| *SN* | *Cylindrospermopsis* | 0 | 0 | 0 | 0 | 0 | 0 | 0 | 0 | 0 | 0 | 0 | 0 | 0 | 0 | 0 | 0 | 0 | 0 | 0 | 0 | 0 | 0 | 0 | 0 | 0 | 0 | 0 | 0 | 0 | 0 | 0 | 0 | 0 | 0 | 0 | 21 | 0 | 0 | 0 |
| *Y* | *Glenodinium* | 0 | 0 | 0 | 0 | 0 | 0 | 0 | 0 | 0 | 0 | 0 | 4 | 0 | 0 | 0 | 0 | 0 | 0 | 0 | 0 | 0 | 0 | 0 | 0 | 10 | 2 | 3 | 0 | 0 | 0 | 0 | 0 | 0 | 1 | 1 | 0 | 0 | 0 | 0 |
| *F* | *Sphaerocystis* | 0 | 0 | 0 | 0 | 0 | 0 | 0 | 0 | 0 | 0 | 0 | 0 | 0 | 0 | 0 | 0 | 0 | 0 | 0 | 0 | 0 | 0 | 0 | 0 | 16 | 0 | 0 | 0 | 0 | 0 | 0 | 0 | 0 | 0 | 0 | 0 | 0 | 0 | 0 |
| *M* | *Encyonema* | 0 | 0 | 0 | 0 | 0 | 0 | 0 | 0 | 0 | 0 | 2 | 0 | 0 | 0 | 0 | 0 | 1 | 4 | 2 | 3 | 0 | 0 | 0 | 0 | 2 | 0 | 0 | 0 | 0 | 0 | 0 | 0 | 0 | 0 | 0 | 0 | 0 | 0 | 0 |
| *LM* | *Ceratium* | 0 | 0 | 0 | 1 | 0 | 0 | 0 | 0 | 0 | 0 | 0 | 0 | 0 | 0 | 0 | 0 | 0 | 0 | 0 | 0 | 0 | 0 | 0 | 0 | 0 | 0 | 2 | 0 | 0 | 0 | 0 | 0 | 0 | 0 | 0 | 0 | 0 | 11 | 0 |
| *X2* | *Chlorogonium* | 0 | 0 | 0 | 0 | 0 | 0 | 0 | 0 | 0 | 0 | 0 | 0 | 0 | 0 | 0 | 0 | 0 | 0 | 0 | 0 | 0 | 0 | 0 | 10 | 0 | 0 | 0 | 1 | 0 | 0 | 0 | 0 | 0 | 0 | 0 | 0 | 0 | 0 | 0 |
| *C* | *Asterionella* | 0 | 0 | 0 | 0 | 0 | 0 | 0 | 0 | 0 | 8 | 0 | 0 | 0 | 0 | 2.33 | 0 | 0 | 0 | 0 | 0 | 0 | 0 | 0 | 0 | 0 | 0 | 0 | 0 | 0 | 0 | 0 | 0 | 0 | 0 | 0 | 0 | 0 | 0 | 0 |
| *TB* | *Ceratoneis* | 0 | 0 | 0 | 0 | 0 | 0 | 0 | 0 | 0 | 0 | 0 | 0 | 0 | 0 | 0 | 0 | 0 | 0 | 0 | 0 | 0 | 0 | 0 | 0 | 4 | 5 | 0 | 0 | 0 | 0 | 0 | 0 | 0 | 0 | 0 | 0 | 0 | 0 | 0 |
| *K* | *Diatoma* | 0 | 0 | 0 | 0 | 0 | 0 | 0 | 0 | 0 | 2 | 0 | 0 | 0 | 1 | 0.67 | 0 | 0 | 3 | 0 | 0 | 2 | 0 | 0 | 0 | 0 | 0 | 0 | 0 | 0 | 0 | 0 | 0 | 0 | 0 | 0 | 0 | 0 | 0 | 0 |
| *S2* | *Cymbopleura* | 0 | 0 | 0 | 0 | 0 | 0 | 0 | 0 | 0 | 0 | 1 | 0 | 0 | 0 | 0 | 0 | 0 | 6 | 0 | 0 | 0 | 0 | 0 | 0 | 0 | 0 | 0 | 0 | 0 | 0 | 0 | 0 | 0 | 0 | 0 | 0 | 0 | 0 | 0 |
| *R* | *Neidium* | 0 | 0 | 0 | 0 | 0 | 0 | 0 | 0 | 0 | 0 | 0 | 1 | 0 | 0 | 0 | 0 | 0 | 0 | 0 | 0 | 0 | 0 | 5 | 0 | 0 | 0 | 0 | 0 | 0 | 0 | 0 | 0 | 0 | 0 | 0 | 0 | 0 | 0 | 0 |
| *X1* | *Ankistrodesmus* | 0 | 0 | 0 | 0 | 0 | 0 | 0 | 0 | 0 | 0 | 0 | 0 | 0 | 0 | 0 | 0 | 0 | 0 | 0 | 0 | 0 | 0 | 0 | 0 | 2 | 3 | 0 | 0 | 0 | 0 | 0 | 0 | 0 | 0 | 0 | 0 | 0 | 0 | 0 |
| *P* | *Closterium* | 0 | 0 | 0 | 0 | 0 | 0 | 0 | 1 | 0 | 1 | 0 | 0 | 0 | 1 | 0 | 0 | 0 | 0 | 0 | 0 | 0 | 0 | 0 | 0 | 0 | 0 | 1 | 0 | 0 | 0 | 0 | 0 | 0 | 0 | 0 | 0 | 1 | 0 | 0 |
| *X2* | *Gyrosigma* | 0 | 0 | 0 | 0 | 0 | 0 | 0 | 0 | 0 | 0 | 1 | 0 | 0 | 0 | 0 | 0 | 0 | 0 | 0 | 0 | 0 | 0 | 0 | 2.5 | 0 | 0 | 0 | 0 | 0 | 0 | 0 | 0 | 0 | 0 | 0 | 0 | 0 | 0 | 1 |
| *B* | *Aulacoseira* | 0 | 0 | 0 | 0 | 0 | 0 | 0 | 0 | 0 | 0 | 0 | 0 | 0 | 0 | 0 | 0 | 0 | 0 | 0 | 0 | 0 | 0 | 0 | 0 | 0 | 0 | 4 | 0 | 0 | 0 | 0 | 0 | 0 | 0 | 0 | 0 | 0 | 0 | 0 |
| *Q* | *Frustulia* | 0 | 0 | 0 | 0 | 0 | 0 | 0 | 0 | 0 | 0 | 0 | 0 | 0 | 0 | 0 | 0 | 0 | 0 | 0 | 0 | 2 | 0 | 0 | 1.25 | 0 | 0 | 0 | 0 | 0 | 0 | 0 | 0 | 0 | 0 | 0 | 0 | 0 | 0 | 0 |
| *W1* | *Amphora* | 0 | 0 | 0 | 0 | 0 | 0 | 0 | 0 | 0 | 0 | 0 | 0 | 0 | 0 | 0 | 0 | 0 | 2 | 1 | 0 | 0 | 0 | 0 | 0 | 0 | 0 | 0 | 0 | 0 | 0 | 0 | 0 | 0 | 0 | 0 | 0 | 0 | 0 | 0 |
| *N* | *Staurastrum* | 0 | 0 | 0 | 0 | 0 | 0 | 0 | 0 | 0 | 0 | 0 | 0 | 0 | 0 | 0 | 0 | 0 | 0 | 0 | 0 | 0 | 0 | 0 | 0 | 0 | 0 | 2 | 0 | 0 | 0 | 0 | 0 | 0 | 0 | 0 | 0 | 0 | 0 | 0 |
| *J* | *Micractinium* | 0 | 0 | 0 | 0 | 0 | 0 | 2 | 0 | 0 | 0 | 0 | 0 | 0 | 0 | 0 | 0 | 0 | 0 | 0 | 0 | 0 | 0 | 0 | 0 | 0 | 0 | 0 | 0 | 0 | 0 | 0 | 0 | 0 | 0 | 0 | 0 | 0 | 0 | 0 |
| *W1* | *Phacus* | 0 | 0 | 0 | 0 | 0 | 0 | 0 | 0 | 0 | 0 | 0 | 0 | 0 | 0 | 0 | 0 | 0 | 0 | 0 | 0 | 0 | 2 | 0 | 0 | 0 | 0 | 0 | 0 | 0 | 0 | 0 | 0 | 0 | 0 | 0 | 0 | 0 | 0 | 0 |
| *J* | *Golenkinia* | 0 | 0 | 0 | 0 | 0 | 0 | 0 | 0 | 1 | 0 | 0 | 0 | 0 | 0 | 0 | 0 | 0 | 0 | 0 | 0 | 0 | 0 | 0 | 0 | 0 | 0 | 0 | 0 | 0 | 0 | 0 | 0 | 0 | 0 | 0 | 0 | 0 | 0 | 0 |
| *J* | *Closteriopsis* | 0 | 0 | 0 | 0 | 0 | 0 | 0 | 0 | 0 | 0 | 0 | 0 | 0 | 0 | 0 | 0 | 0 | 0 | 0 | 0 | 0 | 0 | 0 | 0 | 0 | 0 | 1 | 0 | 0 | 0 | 0 | 0 | 0 | 0 | 0 | 0 | 0 | 0 | 0 |
| *TB* | *Stauroneis* | 0 | 0 | 0 | 0 | 0 | 0 | 0 | 0 | 0 | 0 | 0 | 0 | 0 | 0 | 0 | 0 | 0 | 0 | 1 | 0 | 0 | 0 | 0 | 0 | 0 | 0 | 0 | 0 | 0 | 0 | 0 | 0 | 0 | 0 | 0 | 0 | 0 | 0 | 0 |
